# Novel enterobactin analogues as potential therapeutic chelating agents: Synthesis, thermodynamic and antioxidant studies

**DOI:** 10.1038/srep34024

**Published:** 2016-09-27

**Authors:** Qingchun Zhang, Bo Jin, Zhaotao Shi, Xiaofang Wang, Qiangqiang Liu, Shan Lei, Rufang Peng

**Affiliations:** 1 State Key Laboratory Cultivation Base for Nonmetal Composites and Functional Materials, Southwest University of Science and Technology, Mianyang 621010, China; 2 Department of Chemistry, School of Materials Science and Engineering, Southwest University of Science and Technology, Mianyang 621010, China; 3 Research Center of Laser Fusion, China Academy of Engineering Physics, Mianyang 621010, China

## Abstract

A series of novel hexadentate enterobactin analogues, which contain three catechol chelating moieties attached to different molecular scaffolds with flexible alkyl chain lengths, were prepared. The solution thermodynamic stabilities of the complexes with uranyl, ferric(III), and zinc(II) ions were then investigated. The hexadentate ligands demonstrate effective binding ability to uranyl ion, and the average uranyl affinities are two orders of magnitude higher than 2,3-dihydroxy-*N*^*1*^,*N*^*4*^-bis[(1,2-hydroxypyridinone-6-carboxamide)ethyl]terephthalamide [TMA(2Li-1,2-HOPO)_2_] ligand with similar denticity. The high affinity of hexadentate ligands could be due to the presence of the flexible scaffold, which favors the geometric agreement between the ligand and the uranyl coordination preference. The hexadentate ligands also exhibit higher antiradical efficiency than butylated hydroxyanisole (BHA). These results provide a basis for further studies on the potential applications of hexadentate ligands as therapeutic chelating agents.

The development of civilian energy generation and atomic weapon requires further research on various environment and health issues of uranium^1^. Uranium is introduced into the environment via atomic weapon tests and accidents in nuclear facilities and is then absorbed by humans through ingestion, inhalation, or wounds. The hexavalent uranyl ion [UO_2_^2+^, U(VI)] is the most stable form of this element *in vivo*^2^ and form complexes with chelating agents, such as proteins or carbonates, in the body. Tissues, especially kidney and bones, accumulate uranium for months to years. The radiological accumulation of uranium in tissues causes long-term damage and may induce cancer in the deposition site^3^,^4^,^5^. Uranium with high specific alpha activity can produce harmful free-radicals that activate apoptosis^6^,^7^. Thus, uranium should be excreted from the body by administration of nontoxic chelating agents, which can form stable complexes with the uranyl ion. This treatment results in rapid excretion of the deposited uranium from the blood and target organs, thereby reducing uranium concentration and radiation. In addition, production of harmful free radicals will be inhibited.

The uranyl ion, a hard Lewis acid, exhibits high affinity for hard donor groups. Equatorial pentacoordination or hexacoordination generally occurs between 5- and 6-membered chelated rings with bidentate ligands^8^,^9^,^10^,^11^. In animals, uranium levels can be reduced by injection of bidentate tiron^12^,^13^, moreover, the stability of the U(VI)-catechol complex (log *K*_*ML*_ = 15.9)^14^ indicates that multidentate ligands containing the catechol moiety as binding units are effective for chelation of the uranyl ion. Therefore, a rational approach for design of multidentate sequestering agents for uranyl ion was inspired by enterobactin^15^,^16^, a naturally occurring microbial iron(III)-sequestering agent. A common feature of the design of actinide-sequestering agents is the use of anionic oxygen donors in functional groups, such as catechol from enterobactin. The molecular scaffold should be attached to the catechol moiety in an ortho position relative to phenolate oxygen via amide linkages. The ligands should adopt the correct geometry for metal binding, and the amides contribute to the stability of the iron complex through hydrogen bonding^17^,^18^,^19^. The structure-activity relationship emphasizes that different linker lengths affect the conformation of the complexes^20^. The most potent enterobactin (pFe^3+^ values for Fe^3+^ complex of enterobactin up to 35.5)^21^ is hexadentate, which contains three catechol moieties attached to the molecular scaffold, this feature allows the formation of a coordination cavity suitable for Fe^3+^ 22. However, the ionic radius of uranyl ion (0.95 Å)^23^ is larger than that of Fe^3+^ (0.65 Å)^24^. Therefore, a rational design of hexadentate enterobactin analogue ligands with different molecular scaffolds of flexible alkyl chain lengths must be developed, this design is essential to achieve the coordinative saturation and conformational flexibility of uranyl, thereby allowing the formation of a large coordination cavity suitable for the uranyl ion. To the best of our knowledge, the new hexadentate enterobactin analogues have not been synthesized.

Studies have been performed to obtain a ligand with good chelating ability and antioxidant capacity. The first part of this study involved synthesis of a series of hexadentate enterobactin analogues. The second part involved studying the solution thermodynamic behaviors of these ligands and their complexes with uranyl, iron(III) and zinc(II) ions in aqueous solution. The third part involved evaluation of the antioxidant capacity of the derivatives by 2,2-diphenyl-1-picrylhydrazyl (DPPH·) antioxidant assay^25^,^26^,^27^.

## Results and Discussion

### Synthesis of hexadentate ligands

**Figure 1:**
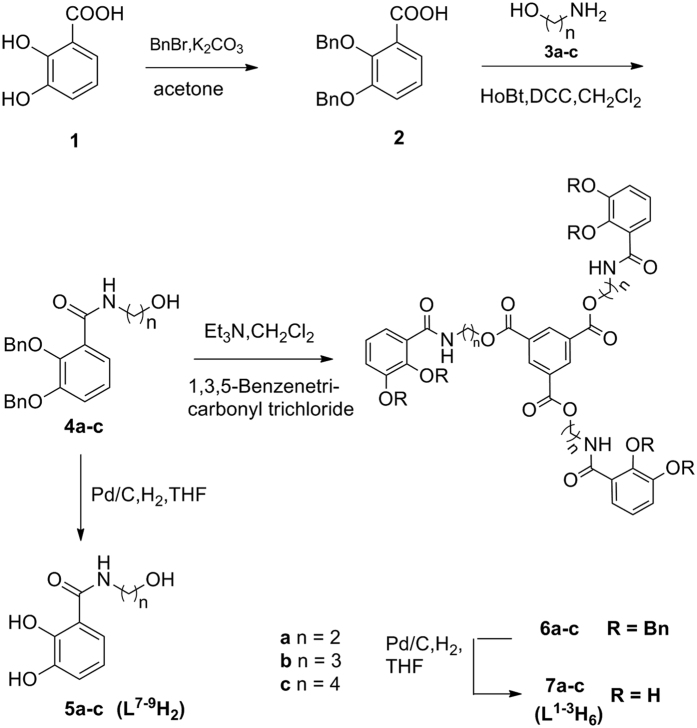
Synthesis of hexadentate enterobactin analogues 7a–c (L^1–3^H_6_) and 5a–c (L^7–9^H_2_).

The preparation of hexadentate enterobactin analogues 7a–c (**L^1–3^H_6_**) is shown in Fig. 1. 2,3-bis(dibenzyloxy)benzonic acid **2** (80%) was generated from commercially available 2,3-bis(hydroxyl)benzonic acid **1**^28^. Aminoalcohol **3a–c** and **2** were condensed using HOBt/DCC to obtain the desired benzamides (**4a–c**) with up to 90% yield^29^. 1,3,5-Benzenetricarbonyl trichloride was then added to benzamides **4a–c** in the presence of Et_3_N in anhydrous CH_2_Cl_2_. The reaction generated benzyl-protected derivatives **6a–c** with up to 71% yield. Deprotection of the hydroxyl groups under typical catalytic hydrogenation conditions with removal of the benzyl group (room temperature, 130 mL/min H_2_, atmospheric pressure, and Pd/C in THF) produced **5a–c** (**L^7–9^H_2_**) and **7a–c** (L^1–3^H_6_) with up to 99% yield.

### Solution thermodynamics

In the presence of dissolved metal ions (M^a+^) and protonated ligands (LH_*i*_, where L is a ligand with *i* removable protons), the pH-dependent metal-ligand complex of general formula M_*m*_L_*l*_H_*h*_ forms according to the equilibrium shown in Eq. 1. The relative amount of each species in solution is determined by Eq. 2, whose rearrangement provides the standard formation constant notation of log *β*_*mlh*_ (Eq. 3). The log *β*_*mlh*_ value describes a cumulative formation constant, and a stepwise formation constant (log *K*) can be calculated from log *β*_*mlh*_ values by Eq. 4. When addressing protonation constants, the stepwise formation constants are commonly reported as log *K*_*i*_^H^ (*i* = 1, 2, 3 …).


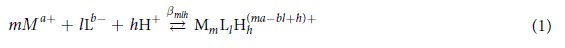



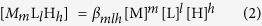



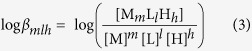



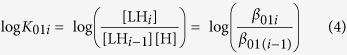


### Proton titration/affinity

**Figure 2:**
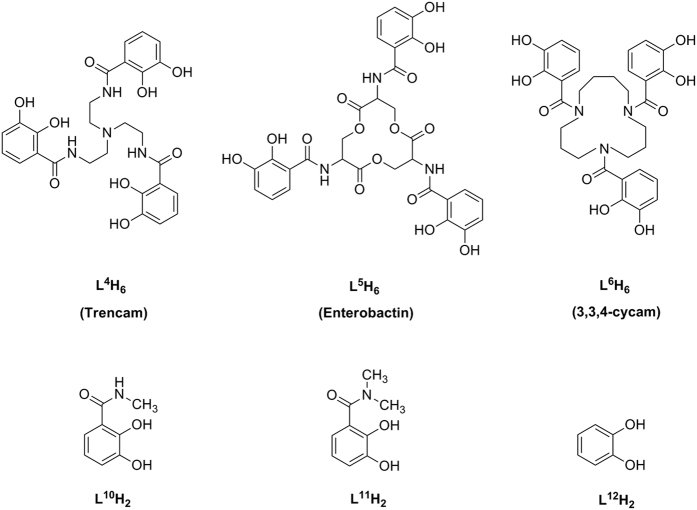
Molecular structure of related compounds.

The protonation constants log *K*_*i*_^H^ (*i* = 1, 2, 3 …) of ligands L^1–3^H_6_ were determined from spectrophotometric titration measurements in aqueous KCl solution an ionic strength of 0.10 M at 298.2 K. The protonation constants for the intermediate L^7–9^H_2_ were also measured and compared with that for ligands L^1–3^H_6_. The determined log *K*_*i*_^H^ values (*i* = 4–6) and estimated values of the related ligands are listed in Table 1 30,31,32 (Fig. 2 for the corresponding structures). The values of L^7–9^H_2_ are listed in Table S1.

**Figure 3:**
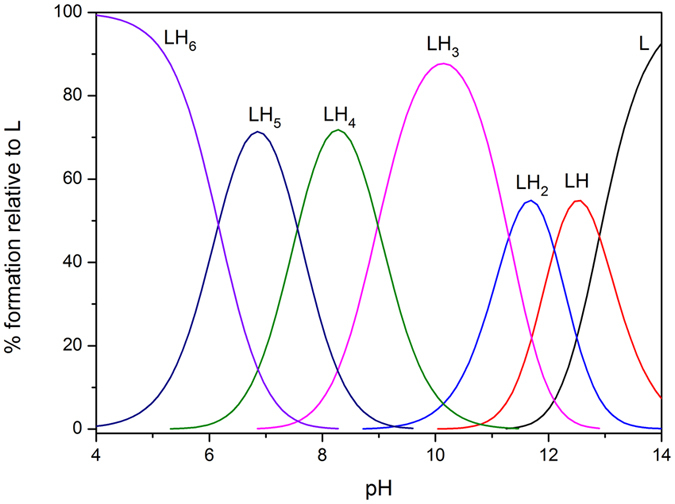
Species distribution curves calculated for the ligand L^1^H_6_, the charge number are omitted for clarity; conditions: [L^1^H_6_] = 2 × 10^−5^ M.

The species distribution diagram of L^1^H_6_ was selected for illustration because it is similar to that of L^1–3^H_6_, as shown in Fig. 3. The species distribution diagram of L^2–3^H_6_ is shown in Figures S1 and S2. These species distribution diagrams were obtained using HySS program^33^. The compounds contain six basic sites form the phenolate oxygen atoms of the catechol moiety. However, only three protonation constants could be accurately determined under our experimental conditions. Indeed, the three values of the first protonation constants of each catechol moiety is very high and cannot be determined by potentiometry.

The catechol derivatives L^7–12^H_2_ differ between the intrinsic acidity of the two dissociable protons of the phenolic oxygen atoms (Table S1). This finding is explained by electronic effects and intramolecular hydrogen bond formation among neighboring amides^17^,^18^,^19^.

The first three protonation constants of L^1–3^H_6_, corresponding to the first protonation of each catechol moiety, cannot be directly determined because of their very high values and the possible oxidation of the ligand at pH = 12.0, as observed in other catecholamide derivatives^34^. The values of the constants are approximately 13.0 for tris- and bis-catechol compounds^31^,^32^,^35^,^36^. Meanwhile, considering the ineluctable statistical factor and the actual practice^30^,^35^, we used the estimated values of log *K*_1_^H^ = 12.9, log *K*_2_^H^ = 12.1 and log *K*_3_^H^ = 11.3 (Table 1).

**Figure 4:**
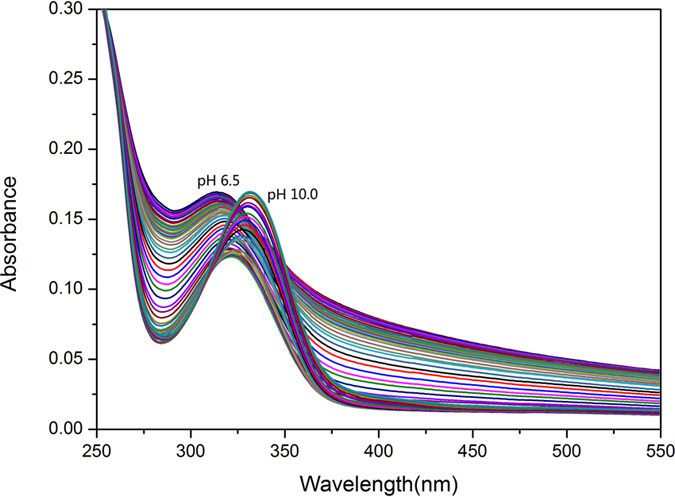
Spectrophotometric titration curves of L^1^H_6_, conditions: [L^1^H_6_] = 2 × 10^−5 ^M; *μ *= 0.10 M KCl; *T *= 298.2 K; pH range = 6.5–10.0; 5.0 vol % methanol aqueous solution.

The spectrophotometric titration curves of L^1–3^H_6_ from pH 6.5 to 10.0 are shown in Fig. 4 and Figures S3 and S4. The initial absorbance at high energy shifts to low energy with increasing pH. The half-peak width gradually narrows, and the intensity of the peak at 330 nm increases upon deprotonation. At pH 10.0, about 90.0% of L^1–3^H_6_ are in the anionic form (L^1–3^H_3_)^3−^ (Fig. 3 and Figures S1 and S2), corresponding each catechol moiety lost one more acidic proton.

The values of log *K*_4_^H^ - log *K*_6_^H^ are ascribed to the three consecutive protonations of the less basic oxygen atoms of the catecholamide dianions with functions similar to amide carbonyl. The average values of the three constants are comparable with the corresponding values for trencam^30^, enterobacin^31^ and 3,3,4-cycam^36^ (Table 1). This finding is in good agreement with the value of log *K*_2_^H^ = 7.31–7.63 for L^7–10^H_2_ (Table S1). The average value of the three more acidic protonation constants of 3,3,4-cycam is 8.59^32^, which is similar to that of L^11^H_2_ (8.42; Table 1 and Table S1). In fact, theoretical calculations, analysis of crystal structures, and experimental potentiometric data for series of catecholamide derivatives indicated that the presence of amide on the catecholamide molecules increased the second protonation constant of the nearby catechol by about one log unit^37^.

### Uranyl titrations/affinity

**Figure 5:**
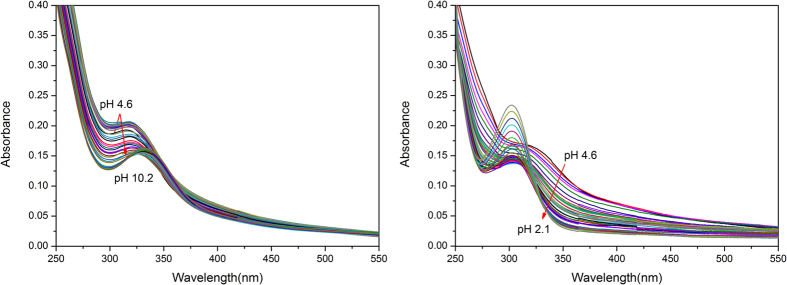
Spectrophotometric titration curves for uranyl with L^1^H_6_, conditions: [UO_2_^2+^] = [L^1^H_6_] = 2 × 10^−5 ^M; *μ *= 0.10 M KCl; *T *= 298.2 K; pH range = 2.1–10.2; 5.0 vol % methanol aqueous solution.

The uranyl affinities of ligands L^1–3^H_6_ were determined by performing spectrophotometric titrations using a 1:1 metal to ligand ratio to avoid the decomposition of free ligand at high pH values. Maintaining this ratio may also ensure the formation of mononuclear complexes. The poor solubility of the uranyl complexes requires 2 × 10^−5^ M analyte and 5 vol % starting methanol for solvating the neutral uranyl complexes during titration. The uranyl titration spectra with L^1–3^H_6_ ligands generally exhibit similar absorption spectra within 250–400 nm, with a maximum absorption within 280–350 nm range and subtle shoulder at long wavelengths (Fig. 5 and Figures S5 and S6). These features resemble those of the free ligands and are attributed to π → π* transitions. The uranyl complexes routinely form [UO_2_(L^1–3^H_4_)] have been generated at pH 4.5, uranyl titration with all ligands displayed increased intensity from pH 4.5 to pH 7.5 for L^1^H_6_, pH 8.1 for L^2^H_6_, and pH 8.4 for L^3^H_6_. These finding indicated the deprotonation of more acidic two protons of the ligands and complexation of the uranyl ion [UO_2_(L^1–3^H_2_)]^2−^. Subsequently, the intensity rapidly decreased until around pH 9.0 and slowly increased until around pH 10.0 with red shift of the absorption peaks. This result revealed the complete deprotonation of the ligands and binding to the uranyl ion [UO_2_(L^1–3^)]^4−^. The acid titrations (pH 4.6 to 2.0) were also carried out for each ligand. The intensity decreased until around pH 3.0 and slowly increased until around pH 2.0 with blue shift of the absorption peaks, which indicated that the protonation of the ligands and binding to the uranyl ion [UO_2_(L^1–3^H_5_)]^+^.

The uranyl titration spectra significantly differ between L^1–3^H_6_ and other reported tetradentate complexes at high pH. The coordinative saturated [UO_2_(L)]^4−^ complexes can form at relatively low pH, and no partial hydrolysis of the uranyl ion occurs with increasing hydroxide concentration. By contrast, the coordination modes of uranyl with tetradentate ligands do not saturate the uranyl coordination plane, hence, the partial hydrolysis of the uranyl ion is predicted to occur at high pH values^38^,^39^,^40^.

The uranyl formation constants log *β*_*mlh*_ for ligands L^1–3^H_6_ are reported in Table 2. A species independent metric is needed to compare uranyl affinities of the bis- and tris-bidentate ligands because log *β*_*mlh*_ values are species dependent. In this regard, pM is the metric employed, where pM = −log[M_free_]. “M_free_” refers to solvated metal ions free of complexation by ligands or hydroxides, high pM corresponds to low concentrations of uncomplexed metal ions in the solution. As a reference index, pUO_2_^2+^ under oceanic conditions is 16.8, which could be due to the high affinity of carbonate for the uranyl ion^41^,^42^. In this study, pUO_2_^2+^ values are calculated using standard conditions of [UO_2_^2+^] = 10^−6^ M and [L] = 10^−5^ M. Typically, these values are reported at physiological pH and can be calculated at any pH upon determination of log *K*_*i*_^H^ and log *β*_*mlh*_ values. The pUO_2_^2+^ values at pH 3.0, 7.4, and 9.0 are listed for ligands L^1–3^H_6_ and related compounds in Table 2.

**Figure 6:**
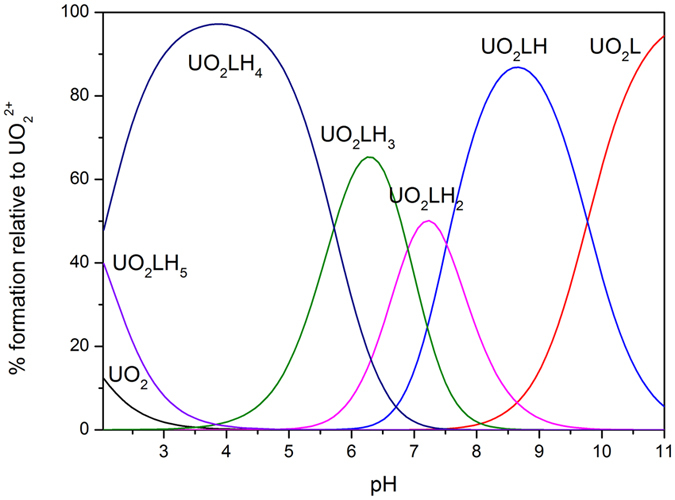
Species distribution curves calculated for uranyl complexes with ligand L^1^H_6_, the charge number are omitted for clarity; conditions: [UO_2_^2+^] = [L^1^H_6_] = 2 × 10^−5 ^M.

The species distribution diagram of L^1^H_6_ was selected for illustration (Fig. 6) because the species distribution diagrams of the uranyl complexes with ligands L^1–3^H_6_ are similar, moreover, the diagrams of ligands L^2–3^H_6_ are shown in Figures S7 and S8.

The pUO_2_^2+^ values of hexadentate L^1–3^H_6_ ligands are significantly higher than those of the tetradentate bis-Me-3,2-HOPO^40^ at all pH values. This finding commonly occurs in L^1–3^H_6_ ligands because of their high denticity. However, the pUO_2_^2+^ values are slightly higher than those of the hexadentate TMA(2Li-1,2-HOPO)_2_^43^, indicating that denticity is not the sole reason. Meanwhile, changes in minor log *K*_*i*_^H^ values are an insignificant factor in determining uranyl affinity in L^1–3^H_6_ ligands, the higher affinity is presumably due to favorable geometric agreement between the ligand and the uranyl coordination preference. The fact that the pUO_2_^2+^ value of L^3^H_6_ is higher than L^1–2^H_6_ indicated that scaffold flexibility favors higher uranyl affinity, as predicted.

### Ferric(III) and zinc(II) ion titrations/affinity

Metal affinity studies have focused on ferric(III) ion, however, the presence of zinc(II) ion in biological systems leads us to evaluate ligands with zinc(II) ion. The ferric(III) and zinc(II) ion affinities of ligands L^1–3^H_6_ were determined through spectrophotometric titrations under the same conditions above. The ferric(III) and zinc(II) ion titration spectra of L^1–3^H_6_ are shown in Figures S9–S14. The species distribution diagrams of ferric(III) and zinc(II) complexes with L^1–3^H_6_ are shown in Figures S15–S20. The ferric(III) and zinc(II) formation constants log *β*_*mlh*_ and pM values at pH 3.0, 7.4, and 9.0 are listed for L^1–3^H_6_ and related compounds in Tables 3 and 4, respectively.

The pFe^3+^ values of hexadentate L^1–3^H_6_ ligands are higher than those of the efficient chelator diethylenetriaminepentaacetic acid (DTPA)^44^ at pH 7.4 but lower than those of enterobactin^21^,^45^ and *N,N’,N”*-tris(2,3-dihydroxybenzoyl)-1,3,5-tris(aminomethyl)benzene (MECAM)^45^, which is an efficient siderophore with high ferric(III) affinity. Enterobactin employs three catechol moieties to tightly encapsulate ferric(III) ion in the hexadentate coordination sphere^46^,^47^. However, the enterobactin analogues L^1-3^H_6_ with longer alkyl chain cannot adequately encapsulate it. This phenomenon could explain the low pFe^3+^ values of L^1–3^H_6_ ligands.

As shown in Table 4, the pZn^2+^ values of hexadentate L^1–3^H_6_ ligands are significantly lower than those of the efficient chelators 1,4,7,10-tetraazacyclododecane-*N,N′,N″,N″′*-tetraacetic acid (DOTA)^44^ and DTPA^44^ at pH 7.4. The low pZn^+^ values are similar to those of hexadentate catechol ligands^48^,^49^,^50^, indicated the formation of catechol derivatives with weak zinc(II) affinity, as predicted.

### Antioxidant activity studies

DPPH· is a radical-generating substance widely used to monitor the free radical scavenging abilities of various antioxidants^25^,^26^,^27^. The assays were carried out in methanol, and the results are expressed as EC_50_, which represents the antioxidant concentration required to decrease the initial DPPH· concentration by 50%. Low EC_50_ values indicate high radical scavenging capacity. This parameter is widely used to measure antioxidant capacity but does not consider the reaction time. The time needed to reach the steady state to the concentration corresponding at EC_50_ (*T*_EC50_) was calculated, and antiradical efficiency (AE) was introduced as a parameter to characterize the antioxidant compounds^27^. AE is determined by Eq. 5.


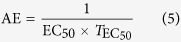


The kinetic curves of phenolic L^1–3^H_6_ with different concentrations are shown in Figures S21–S23. The time of reaction reached a steady state with great difference in the condition of different antioxidant concentrations. The *T*_EC50_ of phenolic L^1–3^H_6_ were obtained by plotting the times at the steady state against the concentration.

The EC_50_ values of phenolic L^1–3^H_6_ were determined from the curves of the percentage of DPPH at the steady state against the molar ratio of antioxidant to DPPH (Figures S24–S26). The EC_50_ and AE values calculated for L^1–3^H_6_ and other compounds are listed in Table 5.

The structures of phenolic derivatives have great influence on the activity^51^,^52^. The phenolic compounds L^1–3^H_6_ exhibit similar antioxidant capacity due to the similar molecular structures. Meanwhile, it is known that the polyphenols are more efficient than monophenols^52^. The DPPH assay results indicated that the hexaphenols L^1–3^H_6_ exihibited lower EC_50_ and shorter *T*_EC50_ values than diphenols catechol^53^ and monophenol BHA^27^, which confers them higher AE values. This result is as expected.

## Conclusions

Coordinative saturation of the uranyl ion is achieved by development of hexadentate enterobactin analogues with different molecular scaffolds containing flexible alkyl chain lengths. The dominance of the hexadentate ligands in uranyl binding is supported by solution phase thermodynamic measurements. The flexible alkyl chain molecular scaffold exhibits conformational flexibility and forms a large coordination cavity suitable for the uranyl ion. The antioxidant capacity is determined by DPPH· assay, the hexadentate ligands are more active than catechol and BHA. Finally, considering the high uranyl affinity of the hexadentate enterobactin analogues, we conclude that these ligands may be more applicable for actinyl ions with larger radius, such as UO_2_^+^ and NpO_2_^+^, in these ions, ligand distortions may be lessened and could be encapsulated.

## Experimental Section

### General

The organic reagents used were pure commercial products from Aladdin. The solvents were purchased from Chengdu Kelong Chemical Reagents Co. Anhydrous CH_2_Cl_2_ was distilled prior to use. The 300–400 mesh silica gels was purchased from Qingdao Hailang Chemical Reagents Co. ^1^H NMR and ^13^C NMR spectra were recorded on Bruker Avance 300, Avance 400, or Avance 600 spectrometer. The FTIR spectra were obtained from Nicolet 380 FTIR spectrophotometer (Thermo Fisher Nicolet, USA) with a resolution of 4 cm^−1^ from 400 cm^−1^ to 4000 cm^−1^. UV-vis spectrophotometer (Thermo Scientific Evolution 201, USA) used had a double-beam light source from 190 nm to 1100 nm. Mass spectral analysis was conducted using Varian 1200 LC/MS.

### 2,3-bis(benzyloxy)benzoic Acid (2)

A solution of 2,3-dihydroxybenzoic acid (10.20 g, 65.9 mmol), benzyl bromide (22.2 g, 130.0 mmol), and K_2_CO_3_ (18.0 g, 130.0 mmol) in acetone (220 mL) was refluxed and stirred for 24 h. After filtration, the solution was concentrated in vacuo to obtain the crude product as clear oil. The crude product was dissolved in methanol (200 mL), and LiOH·H_2_O (360.0 mmol, 15.10 g) was slowly added. The mixture was refluxed and stirred for 3 h. Then, the solution was acidified with 3.0 M HCl to pH 2.0 and filtered to obtain the product **2** as white solid (yield of 80%). ^1^H NMR (400 MHz, CDCl_3_): *δ* (ppm) = 7.50–7.10 (m, 12H, Ar-H), 7.03 (t, *J* = 8.0 Hz, 1H, Ar-H), 5.12 (s, 2H, O-CH_2_-Ar), 5.09 (s, 2H, O-CH_2_-Ar). ^13^C NMR (150 MHz, CDCl_3_): *δ* (ppm) = 165.38 (C=O), 151.54 (ArC), 147.32 (ArC), 136.07 (ArCH), 134.87 (ArCH), 129.51 (ArCH), 129.06 (ArCH), 129.03 (ArCH), 128.77 (ArCH), 128.00 (ArCH), 125.25 (ArCH), 124.67 (ArCH), 123.27 (ArCH), 119.21 (ArCH), 71.77 (CH_2_). FTIR (KBr, cm^−1^): 3100, 2700, 1683, 1035. APCI-MS (m/z): 333.4 [M-H]^−^.

### 2,3-bis(benzyloxy)-*N*-(hydroxyethyl)benzamide (4a)

A solution of 2,3-bis(benzyloxy) benzoic acid **2** (1.67 g, 5.0 mmol), HOBt (0.12 g, 0.9 mmol) and DCC (1.24 g, 6.0 mmol) in CH_2_Cl_2_ (50 mL) was stirred for 30 min at room temperature. Ethanolamine (0.34 g, 5.5 mmol) was added dropwise over 3 min and the mixture stirred 10 h. The solution was filtered to remove the dicyclohexyl urea (DCU). The filtrate was concentrated in vacuo and the residue purified by flash column chromatography to give the product **4a** as clear oil (80%). *R*_*f*_ = 0.4 (volume ratio 2:3 acetone/hexane). ^1^H NMR (300 MHz, CDCl_3_): *δ* (ppm) = 8.30 (br s, 1H, CO-NH), 7.71 (m, 1H, Ar-H), 7.50–7.10 (m, 12H, Ar-H), 5.15 (s, 2H, O-CH_2_-Ar), 5.10 (s, 2H, O-CH_2_-Ar), 3.62 (t, *J* = 5.4 Hz, 2H, CH_2_), 3.40 (m, 2H, CH_2_), 2.87 (br s, 1H, OH). ^13^C NMR (150 MHz, CDCl_3_): *δ* (ppm) = 165.78 (C=O), 150.95 (ArC), 146.15 (ArC), 135.60 (ArC), 127.99 (ArCH), 127.94 (ArCH), 127.52 (ArCH), 126.92 (ArCH), 123.68 (ArCH), 122.49 (ArCH), 116.50 (ArCH), 75.72 (CH_2_), 70.56 (CH_2_), 61.88 (CH_2_), 42.10 (CH_2_). FTIR (KBr, cm^−1^): 3347, 1625, 1577, 1555, 1498, 1028. APCI-MS (m/z): 378.0 [M+H]^+^.

### 2,3-bis(benzyloxy)-*N*-(3-hydroxypropyl)benzamide (4b)

A solution of 2,3-bis(benzyloxy) benzoic acid **2** (1.67 g, 5.0 mmol), HOBt (0.12 g, 0.9 mmol), and DCC (1.24 g, 6.0 mmol) in CH_2_Cl_2_ (50 mL) was stirred for 30 min at room temperature. 3-Amino-1-propanol (0.41 g, 5.5 mmol) was added dropwise over 3 min and the mixture stirred 10 h. The solution was filtered to remove the dicyclohexyl urea (DCU). The filtrate was concentrated in vacuo and the residue purified by flash column chromatography to give the product **4b** as clear oil (85%). *R*_*f*_ = 0.5 (volume ratio 2:3 acetone/hexane). ^1^H NMR (300 MHz, CDCl_3_): δ (ppm) = 8.12 (br s, 1H, CO-NH), 7.72 (m, 1H, Ar-H), 7.50–7.12 (m, 12H, Ar-H), 5.16 (s, 2H, O-CH_2_-Ar), 5.09 (s, 2H, O-CH_2_-Ar), 3.50 (t, *J* = 5.4 Hz, 2H, CH_2_), 3.39 (m, 2H, CH_2_), 1.52 (m, 2H, CH_2_). ^13^C NMR (150 MHz, CDCl_3_): *δ* (ppm) = 165.69 (C=O), 150.94 (ArC), 146.11 (ArC), 135.58 (ArC), 128.08 (ArCH), 127.98 (ArCH), 127.54 (ArCH), 126.89 (ArCH), 123.71 (ArCH), 122.54 (ArCH), 116.43 (ArCH), 75.76 (CH_2_), 70.55 (CH_2_), 57.89 (CH_2_), 34.89 (CH_2_), 31.69 (CH_2_). FTIR (KBr, cm^−1^): 3327, 1635, 1577, 1540, 1452, 1028. APCI-MS (m/z): 392.0 [M+H]^+^.

### 2,3-bis(benzyloxy)-*N*-(4-hydroxybutyl)benzamide (4c)

A solution of 2,3-bis(benzyloxy) benzoic acid **2** (1.67 g, 5.0 mmol), HOBt (0.12 g, 0.9 mmol) and DCC (1.24 g, 6.0 mmol) in CH_2_Cl_2_ (50 mL) was stirred for 30 min at room temperature. 4-Amino-1-butanol (0.49 g, 5.5 mmol) was added dropwise over 3 min and the mixture stirred 10 h. The solution was filtered to remove the dicyclohexyl urea (DCU). The filtrate was concentrated in vacuo and the residue purified by flash column chromatography to give the product **4c** as clear oil (90%). *R*_*f*_ = 0.5 (volume ratio 2:3 acetone/hexane). ^1^H NMR (400 MHz, CDCl_3_): *δ* (ppm) = 8.01 (br s, 1H, CO-NH), 7.74 (m, 1H, Ar-H), 7.50–7.10 (m, 12H, Ar-H), 5.16 (s, 2H, O-CH_2_-Ar), 5.09 (s, 2H, O-CH_2_-Ar), 3.58 (m, 2H, CH_2_), 3.32 (m, 2H, CH_2_), 1.46 (m, 4H, CH_2_-CH_2_). ^13^C NMR (150 MHz, CDCl_3_): *δ* (ppm) = 164.47 (C=O), 150.93 (ArC), 145.99 (ArC), 135.64 (ArC), 127.99 (ArCH), 127.50 (ArCH), 126.90 (ArCH), 123.68 (ArCH), 122.52 (ArCH), 116.17 (ArCH), 75.59 (CH_2_), 70.52 (CH_2_), 61.56 (CH_2_), 38.57 (CH_2_), 29.06 (CH_2_), 25.03 (CH_2_). FTIR (KBr, cm^−1^): 3327, 1635, 1577, 1540, 1452, 1033. APCI-MS (m/z): 406.2 [M+H]^+^.

### 2,3-bis(hydroxy)-*N*-(hydroxyethyl)benzamide (5a)

A mixture of **4a** (0.75 g, 2.0 mmol) and Pd/C (5%) (200 mg) in THF (50 mL) was stirred under H_2_ (130 mL/min) atmosphere for 6 h. The resulting mixture was filtered over celite, evaporated to dryness and dried under vacuum to give **5a** as grey power (yield of 99%). ^1^H NMR (600 MHz, (CD_3_)_2_CO): *δ* (ppm) = 8.15 (br s, 1H, CO-NH), 7.29 (d, *J* = 8.1 Hz, 1H, Ar-H), 6.98 (dd, *J* = 7.8, 1.4 Hz, 1H, Ar-H), 6.73 (t, *J* = 8.0 Hz, 1H, Ar-H), 3.74 (t, *J* = 5.7 Hz, 2H, CH_2_), 3.55 (q, *J* = 5.6 Hz, 2H, CH_2_). ^13^C NMR (150 MHz, (CD_3_)_2_CO): *δ* (ppm) = 170.50 (C=O), 149.70 (ArC), 146.28 (ArC), 118.32 (ArCH), 118.17 (ArCH), 116.84 (ArCH), 114.63 (ArC), 60.20 (CH_2_), 42.09 (CH_2_). FTIR (KBr, cm^−1^): 3370, 2930, 1627, 1593, 1540, 1396, 1338, 1252, 1055. APCI-MS (m/z): 198.2 [M+H]^+^.

### 2,3-bis(hydroxy)-*N*-(3-hydroxypropyl)benzamide (5b)

A mixture of **4b** (0.79 g, 2.0 mmol) and Pd/C (5%) (200 mg) in THF (50 mL) was stirred under H_2_ (130 mL/min) atmosphere for 6 h. The resulting mixture was filtered over celite, evaporated to dryness and dried under vacuum to give **5b** as grey power (yield of 99%). ^1^H NMR (600 MHz, (CD_3_)_2_CO): *δ* (ppm) = 8.26 (br s, 1H, CO-NH), 7.19 (d, *J* = 8.1 Hz, 1H, Ar-H), 6.92 (dd, *J* = 7.8, 1.3 Hz, 1H, Ar-H), 6.67 (t, *J* = 8.0 Hz, 1H, Ar-H), 3.64 (t, *J* = 6.0 Hz, 2H, CH_2_), 3.50 (m, 2H, CH_2_), 1.79 (m, 2H, CH_2_). ^13^C NMR (150 MHz, (CD_3_)_2_CO): *δ* (ppm) = 170.37 (C=O), 149.79 (ArC), 146.37 (ArC), 118.38 (ArCH), 118.22 (ArCH), 116.73 (ArCH), 114.67 (ArC), 59.43 (CH_2_), 36.83 (CH_2_), 31.91 (CH_2_). FTIR (KBr, cm^−1^): 3336, 2932, 1639, 1589, 1547, 1488, 1460, 1334, 1262, 1179, 1070. APCI-MS (m/z): 212.3 [M+H]^+^.

### 2,3-bis(hydroxy)-*N*-(4-hydroxybutyl)benzamide (5c)

A mixture of **4c** (0.81 g, 2.0 mmol) and Pd/C (5%) (200 mg) in THF (50 mL) was stirred under H_2_ (130 mL/min) atmosphere for 6 h. The resulting mixture was filtered over celite, evaporated to dryness and dried under vacuum to give **5c** as a grey power (yield of 99%). ^1^H NMR (600 MHz, (CD_3_)_2_CO): *δ* (ppm) = 8.31 (br s, 1H, CO-NH), 7.28 (d, *J* = 8.1 Hz, 1H, Ar-H), 6.97 (dd, *J* = 7.8, 1.3 Hz, 1H, Ar-H), 6.71 (t, *J* = 8.0 Hz, 1H, Ar-H), 3.62 (m, 2H, CH_2_), 3.45 (m, 2H, CH_2_), 1.72 (m, 2H, CH_2_), 1.63 (m, 2H, CH_2_). ^13^C NMR (150 MHz, (CD_3_)_2_CO): *δ* (ppm) = 170.25 (C=O), 149.82 (ArC), 146.33 (ArC), 118.34 (ArCH), 118.17 (ArCH), 116.80 (ArCH), 114.78 (ArC), 61.27 (CH_2_), 39.17 (CH_2_), 30.01 (CH_2_), 25.51 (CH_2_). FTIR (KBr, cm^−1^): 3409, 3238, 2954, 1644, 1583, 1542, 1474, 1385, 1276, 1236, 1070. APCI-MS (m/z): 225.4 [M+H]^+^.

### 1,3,5-benzenetricarboxylic acid tris[2,3-bis(benzyloxy)-*N*-(hydroxyethyl)benzamide] ester (6a)

A solution of 1,3,5-benzenetricarbonyl trichloride (0.265 g, 1.0 mmol) in CH_2_Cl_2_ (10 mL) was droped in the solution of 2,3-bis(benzyloxy)-*N*-(hydroxyethyl) benzamide **4a** (0.75 g, 2.0 mmol), Et_3_N (2 mL) in CH_2_Cl_2_ (20 mL) under ice bath and vigorous stirring conditions. The mixture stirred at room temperature for 16 h. After evaporation of solvent and the residue purified by flash column chromatography to give the product **6a** as clear oil (yield of 70%). *R*_*f*_ = 0.7 (volume ratio 1:30 methanol/CHCl_3_). ^1^H NMR (600 MHz, CDCl_3_): *δ* (ppm) = 8.62 (s, 3H, Ar-H), 8.19 (t, *J* = 5.8 Hz, 3H, CO-NH), 7.63 (m, 3H, Ar-H), 7.47 (m, 6H, Ar-H), 7.38 (m, 9H, Ar-H), 7.21 (m, 6H, Ar-H), 7.11 (m, 15H, Ar-H), 5.12 (s, 6H, CH_2_), 5.04 (s, 6H, CH_2_), 4.29 (t, *J* = 5.5 Hz, 6H, CH_2_), 3.63 (q, *J* = 5.6 Hz, 6H, CH_2_). ^13^C NMR (150 MHz, CDCl_3_): *δ* (ppm) = 164.39 (C=O), 163.35 (C=O), 150.57 (ArC), 145.60 (ArC), 135.33 (ArC), 135.15 (ArC), 133.54, (ArCH), 129.82 (ArC), 127.64 (ArC), 127.61 (ArCH), 127.56 (ArCH), 127.45 (ArCH), 127.28 (ArCH), 126.77 (ArCH), 126.11 (ArCH), 123.37 (ArCH), 122.06 (ArCH), 75.48 (CH_2_), 70.13 (CH_2_), 63.31 (CH_2_), 37.43 (CH_2_). FTIR (KBr, cm^−1^): 3399, 2973, 2928, 1730, 1647, 1576, 1453, 1263, 1244, 1049, 741, 698. APCI-MS (m/z): 1289.5 [M+H]^+^.

### 1,3,5-benzenetricarboxylic acid tris[2,3-bis(benzyloxy)-*N*-(3-hydroxypropyl)benzamide] ester (6b)

A solution of 1,3,5-benzenetricarbonyl trichloride (0.265 g, 1.0 mmol) in CH_2_Cl_2_ (10 mL) was droped in the solution of 2,3-bis(benzyloxy)-*N*-(3-hydroxypropyl) benzamide **4b** (0.79 g, 2.0 mmol), Et_3_N (2 mL) in CH_2_Cl_2_ (20 mL) under ice bath and vigorous stirring conditions. The mixture stirred at room temperature for 16 h. After evaporation of solvent and the residue purified by flash column chromatography to give the product **6a** as clear oil (yield of 68%). *R*_*f*_ = 0.7 (volume ratio 1:30 methanol/CHCl_3_). ^1^H NMR (600 MHz, CDCl_3_): *δ* (ppm) = 8.67 (s, 3H, Ar-H), 7.98 (t, *J* = 7.1 Hz, 3H, CO-NH), 7.62 (m, 3H, Ar-H), 7.38 (m, 6H, Ar-H), 7.30 (m, 9H, Ar-H), 7.23 (m, 6H, Ar-H), 7.04 (m, 15H, Ar-H), 5.05 (s, 6H, CH_2_), 5.01 (s, 6H, CH_2_), 4.19 (t, *J* = 6.4 Hz, 6H, CH_2_), 3.32 (q, *J* = 6.7 Hz, 6H, CH_2_), 1.74 (m, 6H, CH_2_). ^13^C NMR (150 MHz, CDCl_3_): *δ* (ppm) = 165.28 (C=O), 164.78 (C=O), 151.65 (ArC), 146.86 (ArC), 136.43 (ArC), 134.56 (ArC), 131.15, (ArCH), 128.72 (ArC), 128.71 (ArC), 128.69 (ArCH), 128.26 (ArCH), 127.65 (ArCH), 127.21 (ArCH), 124.35 (ArCH), 123.27 (ArCH), 116.95 (ArCH), 76.48 (CH_2_), 71.26 (CH_2_), 63.46 (CH_2_), 36.59 (CH_2_), 28.53(CH_2_). FTIR (KBr, cm^−1^): 3390, 2929, 1726, 1654, 1575, 1533, 1453, 1262, 1241, 1026, 740, 697. APCI-MS (m/z): 1330.4 [M+H]^+^.

### 1,3,5-benzenetricarboxylic acid tris[2,3-bis(benzyloxy)-*N*-(4-hydroxybutyl)benzamide] ester (6c)

A solution of 1,3,5-benzenetricarbonyl trichloride (0.265 g, 1.0 mmol) in CH_2_Cl_2_ (10 mL) was droped in the solution of 2,3-bis(benzyloxy)-*N*-(3-hydroxypropyl) benzamide **4c** (0.81 g, 2.0 mmol), Et_3_N (2 mL) in CH_2_Cl_2_ (20 mL) under ice bath and vigorous stirring conditions. The mixture stirred at room temperature for 16 h. After evaporation of solvent and the residue purified by flash column chromatography to give the product **6a** as clear oil (yield of 71%). *R*_*f*_ = 0.7 (volume ratio 1:30 methanol/CHCl_3_). ^1^H NMR (600 MHz, CDCl_3_): *δ* (ppm) = 8.72 (s, 3H, Ar-H), 7.90 (t, *J* = 5.6 Hz, 3H, CO-NH), 7.66 (m, 3H, Ar-H), 7.40 (m, 6H, Ar-H), 7.33 (m, 9H, Ar-H), 7.25 (m, 6H, Ar-H), 7.07 (m, 15H, Ar-H), 5.08 (s, 6H, CH_2_), 5.02 (s, 6H, CH_2_), 4.19 (t, *J* = 6.7 Hz, 6H, CH_2_), 3.34 (q, *J* = 6.9 Hz, 6H, CH_2_), 1.61 (m, 6H, CH_2_), 1.36 (m, 6H, CH_2_). ^13^C NMR (150 MHz, CDCl_3_): *δ* (ppm) = 165.12 (C=O), 164.93 (C=O), 151.69 (ArC), 146.85 (ArC), 136.44 (ArC), 134.48 (ArC), 131.39, (ArCH), 128.72 (ArC), 128.71 (ArC), 128.28 (ArCH), 127.67 (ArCH), 127.25 (ArCH), 124.43 (ArCH), 123.36 (ArCH), 116.99 (ArCH), 76.46 (CH_2_), 71.31 (CH_2_), 65.28 (CH_2_), 39.13 (CH_2_), 26.20 (CH_2_), 25.85 (CH_2_). FTIR (KBr, cm^−1^): 3390, 2929, 1726, 1654, 1575, 1533, 1453, 1262, 1241, 1026, 740, 697. APCI-MS (m/z): 1372.8 [M+H]^+^.

### 1,3,5-benzenetricarboxylic acid tris[2,3-bis(hydroxy)-*N*-(hydroxyethyl)benzamide] ester (7a)

A mixture of **6a** (1.29 g, 1.0 mmol) and Pd/C (5%) (200 mg) in THF (50 mL) was stirred under H_2_ (130 mL/min) atmosphere for 6 h. The resulting mixture was filtered over Celite, evaporated to dryness and dried under vacuum to give **7a** as grey power (yield of 99%). ^1^H NMR (600 MHz, (CD_3_)_2_CO): *δ* (ppm) = 8.82 (s, 3H, Ar-H), 7.26 (dd, *J* = 8.1, 1.4 Hz, 3H, Ar-H), 6.98 (dd, *J* = 7.8, 1.4 Hz, 3H, Ar-H), 6.73 (t, *J* = 8.0 Hz, 3H, Ar-H), 4.59 (t, *J* = 5.7 Hz, 6H, CH_2_), 3.87 (m, 6H, CH_2_). ^13^C NMR (150 MHz, (CD_3_)_2_CO): *δ* (ppm) = 170.70 (C=O), 164.48 (C=O), 149.60 (ArC), 146.28 (ArC), 134.20 (ArCH), 131.46 (ArC), 118.50, (ArCH), 118.32 (ArCH), 116.86 (ArCH), 114.56 (ArC), 64.07 (CH_2_), 38.31 (CH_2_). FTIR (KBr, cm^−1^): 3429, 2925, 1723, 1638, 1547, 1460, 1384, 1261, 1042.FTIR (KBr, cm^−1^): 3430, 2955, 1730, 1640, 1544, 1454, 1246, 1157, 1029. APCI-MS (m/z): 748.6 [M+H]^+^.

### 1,3,5-benzenetricarboxylic acid tris[2,3-bis(hydroxy)-*N*-(3-hydroxypropyl)benzamide] ester (7b)

A mixture of **6b** (1.33 g, 1.0 mmol) and Pd/C (5%) (200 mg) in THF (50 mL) was stirred under H_2_ (130 mL/min) atmosphere for 6 h. The resulting mixture was filtered over Celite, evaporated to dryness and dried under vacuum to give **7b** as grey power (yield of 99%). ^1^H NMR (600 MHz, (CD_3_)_2_CO): *δ* (ppm) = 8.75 (s, 3H, Ar-H), 7.20 (dd, *J* = 8.1, 1.3 Hz, 3H, Ar-H), 6.92 (dd, *J* = 7.8, 1.3 Hz, 3H, Ar-H), 6.65 (t, *J* = 8.0 Hz, 3H, Ar-H), 4.51 (t, *J* = 6.2 Hz, 6H, CH_2_), 3.67 (m, 6H, CH_2_), 2.18 (m, 6H, CH_2_). ^13^C NMR (150 MHz, (CD_3_)_2_CO): *δ* (ppm) = 170.43 (C=O), 164.41 (C=O), 149.65 (ArC), 146.25 (ArC), 133.78 (ArCH), 131.45 (ArC), 125.17, (ArCH), 118.29 (ArCH), 118.16 (ArCH), 116.66 (ArCH), 114.52 (ArC), 63.46 (CH_2_), 36.31 (CH_2_), 29.82 (CH_2_). FTIR (KBr, cm^−1^): 3429, 2925, 1723, 1638, 1547, 1460, 1384, 1261, 1042. APCI-MS (m/z): 790.8 [M+H]^+^.

### 1,3,5-benzenetricarboxylic acid tris[2,3-bis(hydroxy)-*N*-(4-hydroxybutyl)benzamide] ester (7c)

A mixture of **6c** (1.37 g, 1.0 mmol) and Pd/C (5%) (200 mg) in THF (50 mL) was stirred under H_2_ (130 mL/min) atmosphere for 6 h. The resulting mixture was filtered over Celite, evaporated to dryness and dried under vacuum to give **7c** as grey power (yield of 99%). ^1^H NMR (600 MHz, (CD_3_)_2_CO): *δ* (ppm) = 8.81 (s, 3H, Ar-H), 7.24 (d, *J* = 8.1 Hz, 3H, Ar-H), 6.94 (dd, *J* = 7.8, 1.3 Hz, 3H, Ar-H), 6.70 (t, *J* = 8.0 Hz, 3H, Ar-H), 4.46 (t, *J* = 6.2 Hz, 6H, CH_2_), 3.52 (m, 6H, CH_2_), 1.91 (m, 12H, CH_2_CH_2_). ^13^C NMR (150 MHz, (CD_3_)_2_CO): *δ* (ppm) = 170.68 (C=O), 164.48 (C=O), 149.62 (ArC), 146.30 (ArC), 134.03 (ArCH), 131.63 (ArC), 118.26, (ArCH), 118.14 (ArCH), 116.66 (ArCH), 114.61 (ArC), 65.14 (CH_2_), 38.69 (CH_2_), 26.00 (CH_2_), 25.76 (CH_2_). FTIR (KBr, cm^−1^): 3410, 2954, 1724, 1639, 1598, 1545, 1459, 1330, 1247, 1167, 1044. APCI-MS (m/z): 832.4 [M+H]^+^.

### Titration solutions and methods

INESA ZDJ-4B automatic potential titrator was used to measure the pH of the experimental solutions. Meanwhile, it was used for incremental additions of base standard solution to the titration cup under N_2_ atmosphere. Titrations were performed in 0.10 M KCl supporting electrolyte. The temperature of the experimental solution was maintained at 298.2 K by an externally thermostat water bath. UV-visible spectra for incremental titrations and batch titrations were recorded on a Thermo Scientific Evolution 201 UV-vis spectrophotometer. Solid reagents were weighed on a Sartorius BT25S analytical balance accurate to 0.01 mg. All titration solutions were prepared using distilled water from Ulupure ULUP-IV ultra water system and degassed by ultrasonic device. Standard solution of 0.10 M KOH and HNO_3_ were purchased from Aladdin. Ligand stock solutions were made by dissolving a weighed amount of ligand accurate to 0.01 mg in 5.0 vol % methanol aqueous solution in volumetric flask. A stock solution of 0.01 M metal ion [uranyl, ferric(III), and zinc(II) ion] were made by dissolving a weighed amount of corresponding metal salt in 5.0 vol % HNO_3_ standard solution. All metal ion titrations were conducted with a 1:1 ligand:metal ratio. Metal-to-ligand ratios were controlled by carefully addition of a ligand solution of known concentration and a metal ion stock solution to the titration cup. All titrations were repeated a minimum of three times.

### Titration date treatment

Spectrophotometric titration data were analyzed using the HypSpec 2014 program^54^, utilizing nonlinear leastsquares regression to determine formation constants. Wavelengths between 250–550 nm were typically used for data refinement except ferric(III) titration. The number of absorbing species to be refined upon was determined by factor analysis within the HypSpec 2014 program^54^. Speciation diagrams were generated by using HySS program^33^ titration simulation software and the protonation and metal complex formation constants determined by potentiometric and spectrophotometric titration experiments.

### Antioxidant assay methods

The antioxidant assay was carried out in dim room. DPPH· methanol solution was made by dissolving a weighed amount of DPPH· in volumetric flask which was wrapped by tinfoil. An aliquot of methanol (0.1 mL), different aliquot stock methanol solution of 5 × 10^−5^ M antioxidant were added to 2.5 mL methanol solution of 6 × 10^−5^ M DPPH·, and the volume adjusted to a final value of 3.0 mL with methanol. Absorbances at 515 nm were measured immediately at 10 s intervals on a Thermo Scientific Evolution 201 UV-vis spectrophotometer until the reaction reached steady state. Five different concentrations were measured for each assay. Then the EC_50_ values were plotted to obtain from graph of the percentage of DPPH· remaining at the steady state against the molar ratio antioxidant to DPPH·. Moreover, the time needed to reach the steady state to EC_50_ concentration (*T*_EC50_) and the AE values were also calculated.

## Additional Information

**How to cite this article**: Zhang, Q. *et al.* Novel enterobactin analogues as potential therapeutic chelating agents: Synthesis, thermodynamic and antioxidant studies. *Sci. Rep.* **6**, 34024; doi: 10.1038/srep34024 (2016).

## Supplementary Material

Supplementary Information

## Figures and Tables

**Table 1:** Protonation constants log *K*_*i*_^H^ of L^1–3^H_6_ and other related compounds.

	Ligand					
	L^1^H_6_^a^	L^2^H_6_^a^	L^3^H_6_^a^	L^4^H_6_^b^	L^5^H_6_^c^	L^6^H_6_^d^
log *K*_1_^H^	12.9^e^	12.9^e^	12.9^e^	12.9^e^	12.9^e^	12.9^e^
log *K*_2_^H^	12.1^e^	12.1^e^	12.1^e^	12.1^e^	12.1^e^	12.1^e^
log *K*_3_^H^	11.3^e^	11.3^e^	11.3^e^	11.26	11.3^e^	11.3^e^
log *K*_4_^H^	8.98 (5)	8.91 (6)	8.86 (8)	8.75	8.55	9.26
log *K*_5_^H^	7.56 (8)	7.52 (4)	7.43 (2)	8.61	7.5	8.65
log *K*_6_^H^	6.16 (7)	6.13 (5)	6.0 (5)	6.71	6.0	7.86
log *K*_7_^H^	—	—	—	5.88	—	—
Average^f^	7.57	7.52	7.43	7.49	7.36	8.59

^a^Determined by spectrophotometric titration: [L^1–3^H_6_] = 2 × 10^−5 ^M; *μ* = 0.10 M KCl; *T* = 298.2 K; pH range = 6.5–10.0; 5.0 vol % methanol aqueous solution.

^b^Ref. 30, *μ* = 0.10 M KNO_3_.

^c^Ref. 31, 5.0 vol % methanol aqueous solution.

^d^Ref. 32, *μ* = 0.10 M KNO_3_.

^e^Estimated values.

^f^Average *K*_*i*_^H^ of the three more acidic catecholamide protonation constants: ∑(log *K*_4_^H^ + log *K*_5_^H^ + log *K*_6_^H^)/3.

**Table 2:** Formation constants log *β*_*mlh*_ and pUO_2_^2+^ values of L^1–3^H_6_ and other related compounds.

ligand	log*β*_11-1_	log*β*_110_	log*β*_111_	log*β*_112_	log*β*_113_	log*β*_114_	log*β*_115_	pUO_2_^2+ ^a		
								pH 3.0	pH 7.4	pH 9.0
L^1^H_6_	—	31.21 (4)	40.98 (3)	48.50 (3)	55.40 (4)	61.12 (3)	63.08 (5)	9.10 (3)	18.88 (1)	23.44 (2)
L^2^H_6_	—	32.72 (5)	42.00 (2)	49.43 (3)	56.71 (2)	62.31 (3)	64.02 (3)	10.41 (2)	20.03 (1)	24.64 (3)
L^3^H_6_	—	33.10 (3)	42.41 (1)	49.68 (4)	57.11 (2)	62.42 (1)	64.21 (3)	10.79 (3)	20.99 (4)	25.00 (1)
TMA(2Li-1,2-HOPO)_2_^b^	—	21.95	26.86	30.79	—	—		6.9	18.2	21.0
PEG-4li-bis-Me- 3,2-HOPO^c^	6.97	13.90	—	—	—	—		8.98	15.39	16.93

^a^pUO_2_^2+^ = −log[UO_2_^2+^_free_], [UO_2_^2+^] = 10^−6 ^M and [L] = 10^−5 ^M.

^b^Ref. 43.

^c^The pUO_2_^2+^ of pH 3.0 and 9.0 are calculated by the log *K*_*i*_^H^ and log *β*_*mlh*_ values of PEG-4li-bis-Me-3,2-HOPO in ref. 40.

**Table 3:** Formation constants log *β*_*mlh*_ and pFe^3+^ values of L^1–3^H_6_ and other related compounds.

ligand	log*β*_110_	log*β*_111_	log*β*_112_	log*β*_113_	log*β*_114_	log*β*_115_	pFe^3+ ^a		
							pH 3.0	pH 7.4	pH 9.0
L^1^H_6_	41.66 (4)	49.60 (1)	57.26 (3)	63.56 (3)	66.12 (5)	67.18 (6)	14.39 (6)	27.58 (2)	33.06(1)
L^2^H_6_	40.81 (6)	48.74 (4)	56.34 (2)	62.48 (3)	65.01 (7)	66.05 (8)	13.62 (7)	26.78 (2)	32.24 (3)
L^3^H_6_	40.13 (8)	48.25 (5)	56.02 (7)	61.62 (2)	64.08 (7)	65.06 (7)	13.02 (6)	26.47 (4)	31.61 (1)
MECAM^b^	43.0	50.2	56.23	60.73	64.53	—	13.20	29.40	34.56
Enterobactin^b^	49.0	53.95	57.47	59.97	—	—	12.28	35.50	40.52
DTPA^c^	—	—	—	—	—	—	—	24.60	—

^a^pFe^3+^ = −log[Fe^3+^_free_], [Fe^3+^] = 10^−6 ^M and [L] = 10^−5 ^M.

^b^The pFe^3+^ of pH 3.0 and 9.0 are calculated by the log *K*_*i*_^H^ and log *β*_*mlh*_ values of enterobactin in refs 31 and 45.

^c^Ref. 44.

**Table 4:** Formation constants log *β*_*mlh*_ and pZn^2+^ values of L^1–3^H_6_ and other related compounds.

ligand	log*β*_110_	log*β*_111_	log*β*_112_	log*β*_113_	pZn^2+ ^a		
					pH 3.0	pH 7.4	pH 9.0
L^1^H_6_	14.28 (3)	23.01 (4)	31.30 (5)	34.17 (7)	6.0	6.0	6.24 (2)
L^2^H_6_	15.30 (6)	24.21 (4)	32.1 (2)	35.4 (3)	6.0	6.0 (1)	7.01 (2)
L^3^H_6_	14.81 (8)	23.46 (5)	31.56 (7)	34.52 (2)	6.0	6.0 (1)	6.55 (1)
DOTA^b^	—	—	—	—	—	17.9	—
DTPA^b^	—	—	—	—	—	14.8	—

^a^pZn^2+^ = −log[Zn^2+^_free_], [Zn^2+^] = 10^−6 ^M and [L] = 10^−5 ^M.

^b^Ref. 44.

**Table 5:** Effective concentration (EC_50_) and antiradical efficiency (AE) obtained with DPPH· assay.

Compounds	EC_50_ (mol AH^a^/mol DPPH·)	*T*_EC50_ (min)	AE (×10^−3^)
L^1^H_6_	0.073	60.0	228 ± 5
L^2^H_6_	0.065	60.0	256 ± 10
L^3^H_6_	0.070	65.0	220 ± 8
Catechol^b^	0.09	122.1	91
BHA^c^	0.203	103.9	48

^a^Antioxidant.

^b^Ref. 53.

^c^Ref. 27.

## References

[b1] Allard B., Olofsson U. & Torstenfelt B. Environmental actinide chemistry. Inorg. Chim. Acta 94, 205–221 (1984).

[b2] Hamilton J. G. The metabolic properties of the fission products and actinide elements. Rev. Mod. Phys. 20, 718–728 (1948).

[b3] Galle P. Toxiques Nucléaires 185–205 (Masson, 1997).

[b4] Brugge D., de Lemos J. L. & Oldmixon B. Exposure pathways and health effects associated with chemical and radiological toxicity of natural uranium: a review. Rev. Environ. Health 20, 177–193 (2005). 16342416 10.1515/reveh.2005.20.3.177

[b5] Durbin P. W. In The Chemistry of the Actinide and Transactinide Elements 3rd edn, vol. 5 (eds Morss L. R. *et al.*) 3329 (Springer Science & Business Media, 2006).

[b6] Pellmar T. C., Keyser D. O., Emery C. & Hogan J. B. Electrophysiological changes in hippocampal slices isolated from rats embedded with depleted uranium fragments. Neurotoxicology 20, 785–792 (1999). 10591514

[b7] Periyakaruppan A., Kumar F., Sarkar S., Sharma C. S. & Ramesh G. T. Uranium induces oxidative stress in lung epithelial cells. Arch. Toxicol 81, 389–395 (2007). 17124605 10.1007/s00204-006-0167-0 PMC2740373

[b8] Xu X. T. *et al.* UO_2_^2+^-amino hybrid materials: structural variation and photocatalysis properties. CrystEngComm 17, 642–652 (2015).

[b9] Sather A. C., Berryman O. B., Moore C. E. & Jr J. R. Uranyl ion coordination with rigid aromatic carboxylates and structural characterization of their complexes. Chem. Commun. 49, 6379–6381 (2013). 10.1039/c3cc43358g 23752768

[b10] Frasson E., Bombieri G. & Panattoni C. Stereochemistry of uranyl acetylacetonate monohydrate. Coord. Chem. Rev. 1, 145–150 (1966).

[b11] Harrowfield J. M., Kepert D. L., Patrick J. M., White A. H. & Lincoln S. F. Crystal structure of pentakis (dimethyl sulphoxide-O) dioxouranium (VI) bis(perchlorate). J. Chem. Soc., Dalton Trans. 2, 393–396 (1983).

[b12] Domingo J. L., Ortega A., Llobet J. M., Paternain J. L. & Corbella J. The effects of repeated parenteral administration of chelating agents on the distribution and excretion of uranium. Res. Commun. Chem. Pathol. Pharmacol. 64, 161–164 (1989). 2748997

[b13] Stradling G. N., Gray S. A., Moody J. C. & Ellender M. Efficacy of tiron for enhancing the excretion of uranium from the rat. Hum. Exp. Toxicol. 10, 195–198 (1991). 1678949 10.1177/096032719101000308

[b14] Sylwester E. R., Allen P. G., Dharmawardana U. R. & Sutton M. Structural studies of uranium and thorium complexes with 4,5-dihydroxy-3,5-benzenesdisulfonate (Tiron) at low and neutral pH by X-ray absorption spectroscopy. Inorg. Chim. 40, 2835–2841 (2001). 10.1021/ic001223t 11375702

[b15] Harris W. R. *et al.* Coordination chemistry of microbial iron transport compounds. 19. Stability constants and electrochemical behavior of ferric enterobactin and model complexes. J. Am. Chem. Soc. 101, 6097–6104 (1979).

[b16] Harris W. R., Carrano C. J. & Raymond K. N. Spectrophotometric determination of the proton-dependent stability constant of ferric enterobactin. J. Am. Chem. Soc. 101, 2213–2214 (1979).

[b17] Huang S. P., Franz K. J., Olmstead M. M. & Fish R. H. Synthetic and structural studies of a linear bis-catechol amide, *N, N’*-bis(2,3-dihydroxybenzoyl)-1,7-diazaheptane (5-LICAM), and its complexes with Ni^2+^ and Co^2+^: utilization of a polymer-supported, sulfonated analog, 5-LICAMS, as a biomimetic ligand for divalent metal ion removal from aqueous solution. Inorg. Chim. 34, 2820–2825 (1995).

[b18] Xu J., Kullgren B., Durbin P. W. & Raymond K. N. Specific sequestering agents for the actinides. 28. Synthesis and initial evaluation of multidentate 4-carbamoyl-3-hydroxy-1-methyl-2(1H)-pyridinone ligands for *in vivo* plutonium (IV) chelation. J. Med. Chem. 38, 2606–2614 (1995). 7629800 10.1021/jm00014a013

[b19] Xu J., O’Sulliva B. & Raymond K. N. Hexadentate Hydroxypyridonate Iron Chelators Based on TREN-Me-3, 2-HOPO: Variation of Cap Size. Inorg. Chim. 41, 6731–6742 (2002). 10.1021/ic025610+ 12470069

[b20] Xu J. & Raymond K. N. Uranyl sequestering agents: correlation of properties and efficacy with structure for UO_2_^2+^ complexes of linear tetradentate 1-methyl-3-hydroxy-2(1H)-pyridinone ligands. Inorg. Chem. 38, 308–315 (1999).

[b21] Harris W. R., Raymond K. N. & Weitl F. L. Ferric ion sequestering agents. 6. The spectrophotometric and potentiometric evaluation of sulfonated tricatecholate ligands. J. Am. Chem. Soc. 103, 2667–2675 (1981).

[b22] Raymond K. N. & Smith W. L. In Structure and Bonding *vol. 43* (ed. Goodenough J. B.) (Springer-Verlag, 1981).

[b23] Dean N. E., Hancock R. D., Cahill C. L. & Frisch M. Affinity of the highly preorganized ligand PDA (1,10-phenanthroline-2,9-dicarboxylic acid) for large metal ions of higher charge. A crystallographic and thermodynamic study of PDA complexes of thorium (IV) and the uranyl (VI) ion. Inorg. Chim. 47, 2000–2010 (2008). 10.1021/ic701574j 18284190

[b24] Raymond K. N., Freeman G. E. & Kappel M. J. Actinide-specific complexing agents: their structural and solution chemistry. Inorg. Chim. Acta 94, 193–204 (1984).

[b25] Romano C. S., Abadi K., Repetto V., Vojnov A. A. & Moreno S. Synergistic antioxidant and antibacterial activity of rosemary plus butylated derivatives. Food Chem. 115, 456–461 (2009).

[b26] Sharma O. P. & Bhat T. K. DPPH antioxidant assay revisited. Food Chem. 113, 1202–1205 (2009).

[b27] Concepción S. M., Larrauri J. A. & Calixto F. S. A procedure to measure the antiradical efficiency of polyphenols. J. Sci. Food Agric. 76, 270–276 (1998).

[b28] Laursen B., Denieul M. P. & Skrydstrup T. Formal total synthesis of the PKC inhibitor, balanol: preparation of the fully protected benzophenone fragment. Tetrahedron 58, 2231–2238 (2002).

[b29] Gardner R. A., Kinkade R., Wang C. & Phanstiel IV O. Total Synthesis of petrobactin and its homologues as potential growth stimuli for marinobacter hydrocarbonoclasticus, an Oil-Degrading Bacteria. J. Org. Chem. 69, 3530–3537 (2004). 15132566 10.1021/jo049803l

[b30] Rodgers S. J., Lee C. W., Ng C. Y. & Raymond K. N. Ferric ion sequestering agents. 15. Synthesis, solution chemistry, and electrochemistry of a new cationic analog of enterobactin. Inorg. Chim. 26, 1622–1625 (1987).

[b31] Loomis L. D. & Raymond K. N. Solution equilibria of enterobactin and metal-enterobactin complexes. Inorg. Chim. 30, 906–911 (1991).

[b32] Harris W. H. & Raymond K. N. Ferric ion sequestering agents. 3. The spectrophotometric and potentiometric evaluation of two new enterobactin analogs: 1,5,9-*N,N’,N”*-tris(2,3-dihydroxybenzoyl) cyclotriazatridecane and 1,3,5-*N,N’,N”*-tris(2,3-dihydroxybenzoyl) triaminomethylbenzene. J. Am. Chem. Soc. 101, 6534–6541 (1979).

[b33] Alderighi L. *et al.* Hyperquad simulation and speciation (HySS): a utility program for the investigation of equilibria involving soluble and partially soluble species. Coord. Chem. Rev. 184, 311–318 (1999).

[b34] Imbert D. *et al.* Synthesis and iron (III) complexing ability of CacCAM, a new analog of enterobactin possessing a free carboxylic anchor arm. Comparative studies with TRENCAM. New J. Chem. 24, 281–288 (2000).

[b35] Harris W. R., Raymond K. N. & Weitl F. L. Ferric ion sequestering agents. 6. The spectrophotometric and potentiometric evaluation of sulfonated tricatecholate ligands. J. Am. Chem. Soc. 103, 2667–2675 (1981).

[b36] Hou Z., Stack T. D. P., Sunderland C. J. & Raymond K. N. Enhanced iron (III) chelation through ligand predisposition: syntheses, structures and stability of tris-catecholate enterobactin analogs. Inorg. Chim. Acta 263, 341–355 (1997).

[b37] Hay B. P., Dixon D. A., Vargas R., Garza J. & Raymond K. N. Structural criteria for the rational design of selective ligands. 3. Quantitative structure-stability relationship for iron (III) complexation by tris-catecholamide siderophores. Inorg. Chim. 40, 3922–3935 (2001). 10.1021/ic001380s 11466050

[b38] Szigethy G. & Raymond K. N. Influence of linker geometry on uranyl complexation by rigidly linked bis(3-hydroxy-*N*-methyl-pyridin-2-one). Inorg. Chim. 49, 6755–6765 (2010). 10.1021/ic1007878 20575583

[b39] Xu J. & Raymond K. N. Uranyl sequestering agents: correlation of properties and efficacy with structure for UO_2_^2+^ complexes of linear tetradentate 1-methyl-3-hydroxy-2(1H)-pyridinone ligands. Inorg. Chem. 38, 308–315 (1999).

[b40] Szigethy G. & Raymond K. N. The influence of linker geometry in Bis(3-hydroxy-*N*-methyl-pyridin-2-one) ligands on solution phase uranyl affinity. Chem.-Eur. J. 17, 1818–1827 (2011). 21274933 10.1002/chem.201002372

[b41] Schwochau K. Topics in Current Chemistry 91–133 (Spinger, 1984).

[b42] Martell A. E. & Smith R. M. In Critical Stability Constants, vol. 5 (Plenum, 1977).

[b43] Szigethy G. & Raymond K. N. Hexadentate terephthalamide (bis-hydroxypyridinone) ligands for uranyl chelation: structural and thermodynamic consequences of ligand variation. J. Am. Chem. Soc. 133, 7942–7956 (2011). 21542587 10.1021/ja201511u

[b44] Santos M. A., Gama S., Gano L., Cantinho G. & Farkas E. A new bis(3-hydroxy-4-pyridinone)-IDA derivative as a potential therapeutic chelating agent. Synthesis, metal-complexation and biological assays. Dalton Trans. 3772–3781 (2004). 15510305 10.1039/B409357G

[b45] Hou Z., Stack T. D. P., Sunderland C. J. & Raymond K. N. Enhanced iron(III) chelation through ligand predisposition: syntheses, structures and stability of tris-catecholate enterobactin analogs. Inorg. Chim. Acta 263, 341–355 (1997).

[b46] Isied S. S., Kuo G. & Raymond K. N. Coordination isomers of biological iron transport compounds. V. The preparation and chirality of the chromium (III) enterobactin complex and model tris(catechol) chromium (III) analogues. J. Am. Chem. Soc. 98, 1763–1767 (1976). 130395 10.1021/ja00423a021

[b47] Scarrow R. C., Ecker D. J., Ng C., Liu S. & Raymond K. N. Iron (III) coordination chemistry of linear dihydroxyserine compounds derived from enterobactin. Inorg. Chim. 30, 900–906 (1991).

[b48] Guerra K. P. & Delgado R. Homo-and heterodinuclear complexes of the tris(catecholamide) derivative of a tetraazamacrocycle with Fe^3+^, Cu^2+^ and Zn^2+^ metal ions. Dalton Trans. 4, 539–550 (2008). 18185872 10.1039/b712916e

[b49] Kappel M. J. & Raymond K. N. Ferric ion sequestering agents. 10. Selectivity of sulfonated poly(catechoylamides) for ferric ion. Inorg. Chim. 21, 3437–3442 (1982).

[b50] Biaso F., Baret P., Pierre J. L. & Serratrice G. Comparative studies on the iron chelators O-TRENSOX and TRENCAMS: selectivity of the complexation towards other biologically relevant metal ions and Al^3+^. J. Inorg. Biochem. 89, 123–130 (2002). 11931972 10.1016/s0162-0134(01)00401-9

[b51] Cuvelier M. E., Richard H. & Berset C. Comparison of the antioxidative activity of some acid-phenols: structure-activity relationship. Biosci. Biotechnol. Biochem. 56, 324–325 (1992).

[b52] Shahidi F., Janitha P. K. & Wanasundara P. D. Phenolic antioxidants. Crit. Rev. Food Sci. Nutr. 32, 67–103 (1992). 1290586 10.1080/10408399209527581

[b53] Bortolomeazzi R., Sebastianutto N., Toniolo R. & Pizzariello A. Comparative evaluation of the antioxidant capacity of smoke flavouring phenols by crocin bleaching inhibition, DPPH radical scavenging and oxidation potential. Food Chem. 100, 1481–1489 (2007).

[b54] Gans P., Sabatini A. & Vacca A. Determination of equilibrium constants from spectrophometric data obtained from solutions of known pH: the program pHab. Ann. Chim. 89, 45–49 (1999).

