# Supplementary material for: Novel enterobactin analogues as potential therapeutic chelating agents: Synthesis, thermodynamic and antioxidant studies
Source: Sci Rep. 2016 Sep 27;6:34024. doi: 10.1038/srep34024 (PMC5037427; doi:10.1038/srep34024)
Supplement: Supplementary Information [file srep34024-s1.pdf]

Supplementary Information

Novel enterobactin analogues as a potential therapeutic chelating agent. Synthesis, thermodynamic and antioxidant studies

Qingchun Zhang,[a] Bo Jin,[a,b,]* Zhaotao Shi,[b] Xiaofang Wang,[a] Qiangqiang Liu,[a,c] Shan Lei,[a] Rufang Peng,[a,b,]*

[a] State Key Laboratory Cultivation Base for Nonmetal Composites and Functional Materials, Southwest University of Science and Technology, Mianyang 621010, China

[b] Department of Chemistry, School of Materials Science and Engineering, Southwest University of Science and Technology, Mianyang 621010, China

[c] Research Center of Laser Fusion, China Academy of Engineering Physics, Mianyang 621010, China

Table S1 Protonation constants (log K_i^H) of $L^{7-9}H_2$ and other related compounds.

	Ligand					
	L^7H_2 *a*	L^8H_2 *a*	L^9H_2 *a*	$L^{10}H_2$ *b*	$L^{11}H_2$ *c*	$L^{12}H_2$ *d*
log K_1^H	11.60	11.40	11.37	11.20	12.10	13.00
log K_2^H	7.63	7.50	7.31	7.50	8.42	9.24

a Determined from a combination of potentiometric and spectrophotometric titrations: $[L^{7-9}H_2] = 3.5 \times 10^{-4}$ M; μ = 0.10 M KCl; T = 298.2 K. *b* Ref. 1. *c* Ref. 2. *d* Ref. 3.

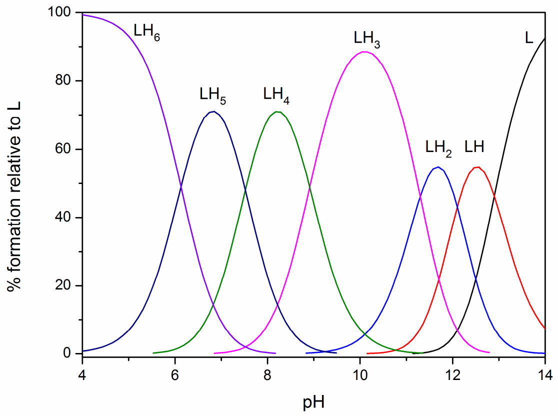

Fig. S1 Species distribution curves calculated for the ligand L^2H_6, the charge number are omitted for clarity, conditions: $[L^2H_6] = 2 \times 10^{-5}$ M.

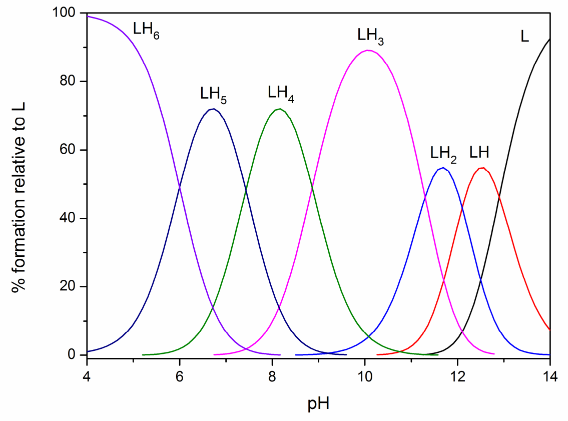

Fig. S2 Species distribution curves calculated for the ligand L^3H_6, the charge number are omitted for clarity, conditions: $[L^3H_6] = 2 \times 10^{-5}$ M.

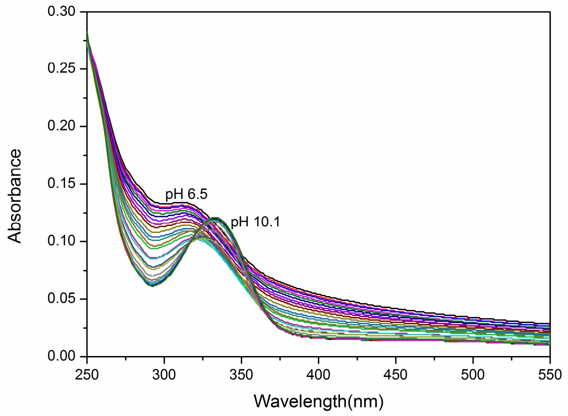

Fig. S3 Spectrophotometric titration curves of L^2H_6, conditions: $[L^2H_6] = 2 \times 10^{-5}$ M; $\mu = 0.10$ M KCl; $T = 298.2$ K; pH range = 6.5-10.1; 5.0 vol % methanol aqueous solution.

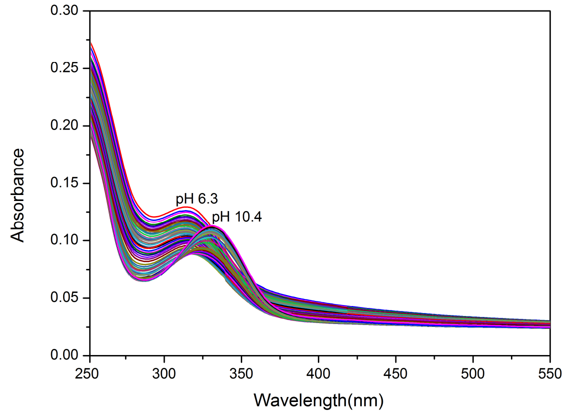

Fig. S4 Spectrophotometric titration curves of L^3H_6, conditions: $[L^3H_6] = 2 \times 10^{-5}$ M; $\mu = 0.10$ M KCl; $T = 298.2$ K; pH range = 6.3-10.4; 5.0 vol % methanol aqueous solution.

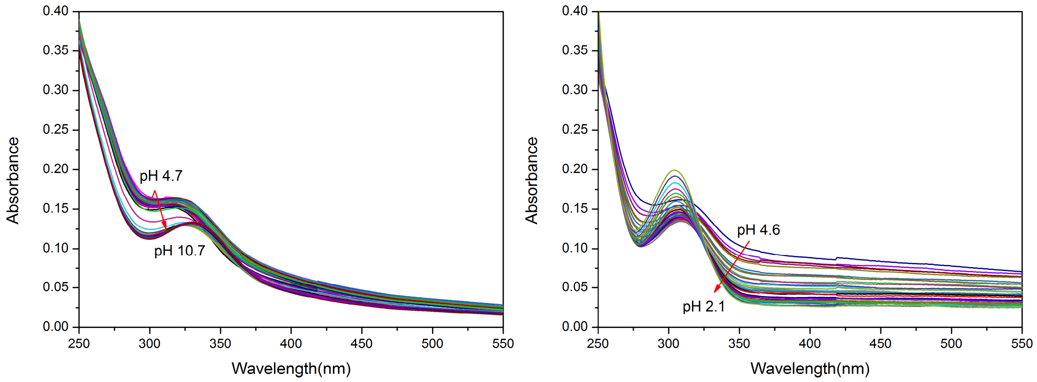

Fig. S5 Spectrophotometric titration curves for uranyl with L^2H_6, conditions: $[UO_2^{2+}] = [L^2H_6] = 2 \times 10^{-5}$ M; $\mu = 0.10$ M KCl; $T = 298.2$ K; pH range = 2.1-10.7; 5.0 vol % methanol aqueous solution.

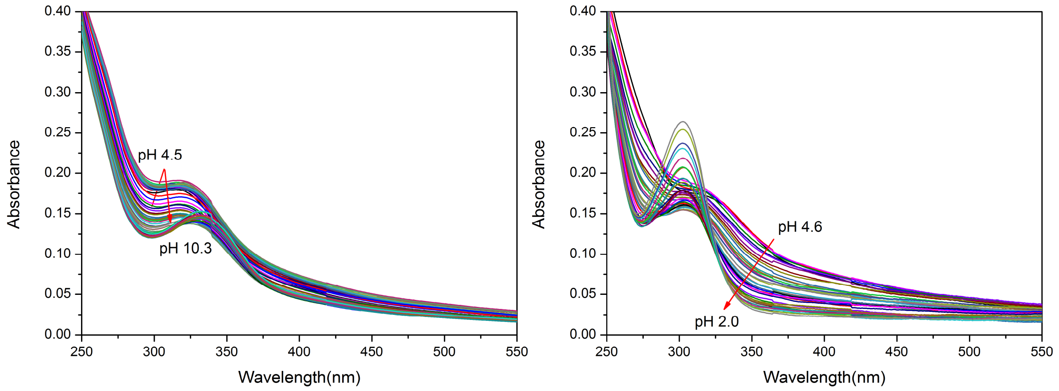

Fig. S6 Spectrophotometric titration curves for uranyl with L^3H_6, conditions: $[UO_2^{2+}] = [L^3H_6] = 2 \times 10^{-5}$ M; $\mu = 0.10$ M KCl; $T = 298.2$ K; pH range = 2.0-10.3; 5.0 vol % methanol aqueous solution.

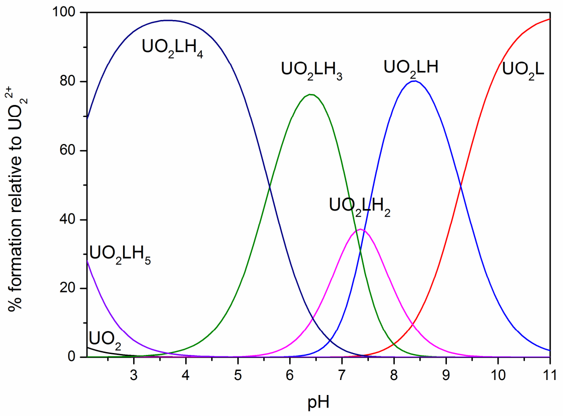

Fig. S7 Species distribution curves calculated for the uranyl complexes with ligand L^2H_6, the

charge number are omitted for clarity, conditions: $[UO_2^{2+}] = [L^2H_6] = 2 \times 10^{-5}$ M.

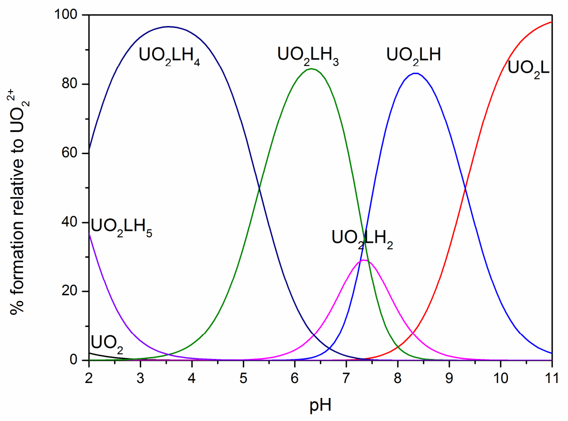

Fig. S8 Species distribution curves calculated for the uranyl complexes with ligand L^3H_6, the charge number are omitted for clarity, conditions: $[UO_2^{2+}] = [L^2H_6] = 2 \times 10^{-5}$ M.

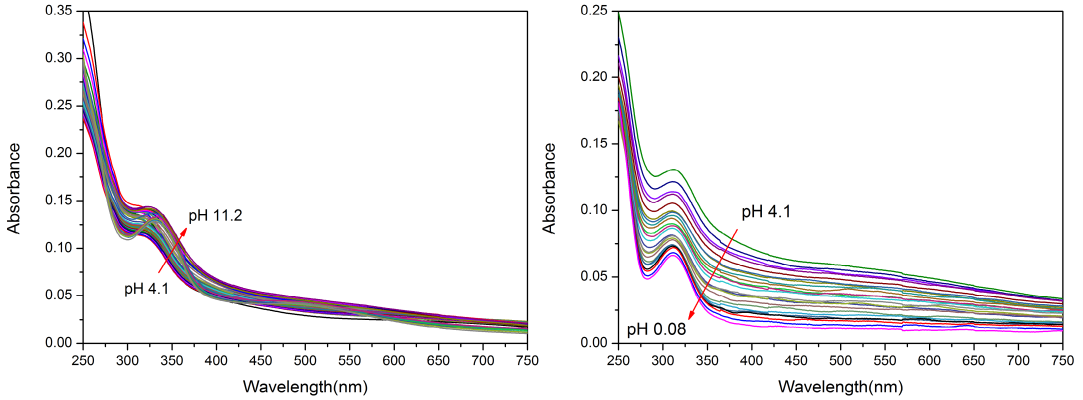

Fig. S9 Spectrophotometric titration curves for ferric(III) with L^1H_6, conditions: $[Fe^{3+}] = [L^1H_6] = 2 \times 10^{-5}$ M; $\mu = 0.10$ M KCl; $T = 298.2$ K; pH range = 0.08-11.2; 5.0 vol % methanol aqueous solution.

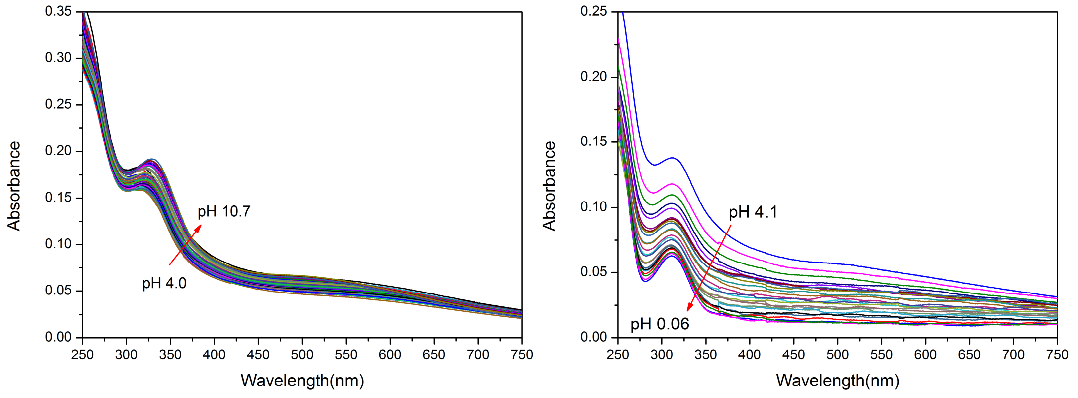

Fig. S10 Spectrophotometric titration curves for ferric(III) with L^2H_6, conditions: $[Fe^{3+}] = [L^2H_6]$

$= 2 \times 10^{-5}$ M; $\mu = 0.10$ M KCl; $T = 298.2$ K; pH range = 0.06-10.7; 5.0 vol % methanol aqueous solution.

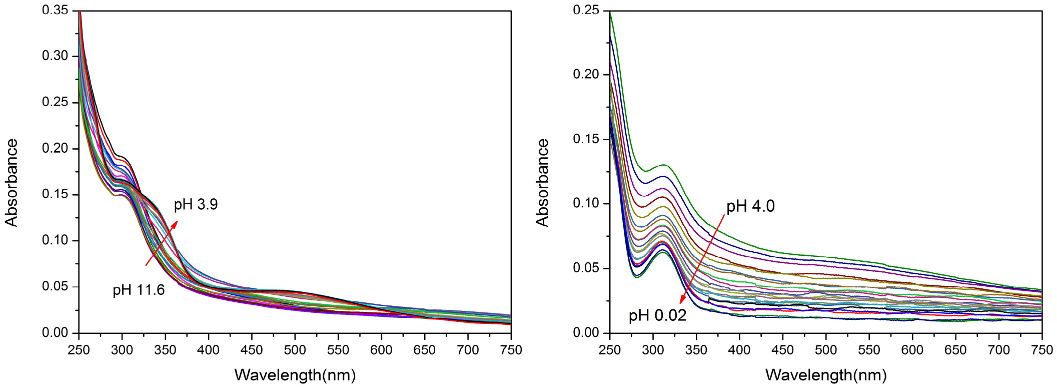

Fig. S11 Spectrophotometric titration curves for ferric(III) with L^3H_6, conditions: $[Fe^{3+}] = [L^3H_6] = 2 \times 10^{-5}$ M; $\mu = 0.10$ M KCl; $T = 298.2$ K; pH range = 0.02-11.6; 5.0 vol % methanol aqueous solution.

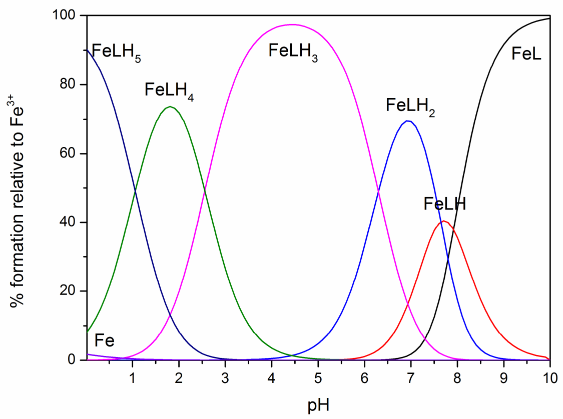

Fig. S12 Species distribution curves calculated for the ferric(III) complexes with ligand L^1H_6, the charge number are omitted for clarity, conditions: $[Fe^{3+}] = [L^1H_6] = 2 \times 10^{-5}$ M.

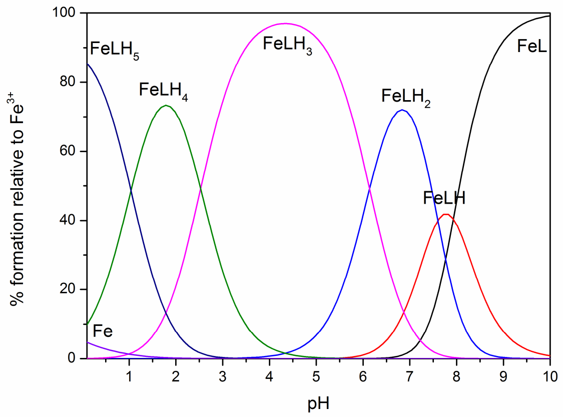

Fig. S13 Species distribution curves calculated for the ferric(III) complexes with ligand L^2H_6, the charge number are omitted for clarity, conditions: $[Fe^{3+}] = [L^2H_6] = 2 \times 10^{-5}$ M.

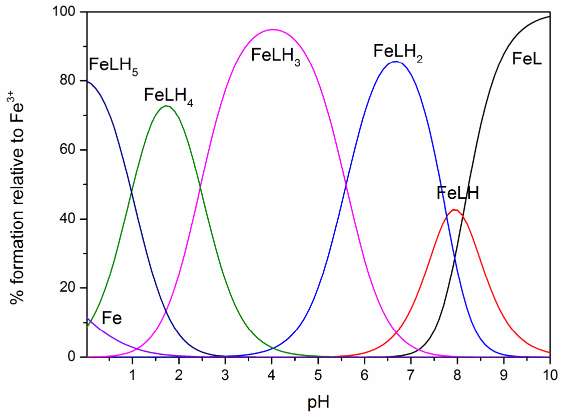

Fig. S14 Species distribution curves calculated for the ferric(III) complexes with ligand L^3H_6, the charge number are omitted for clarity, conditions: $[Fe^{3+}] = [L^3H_6] = 2 \times 10^{-5}$ M.

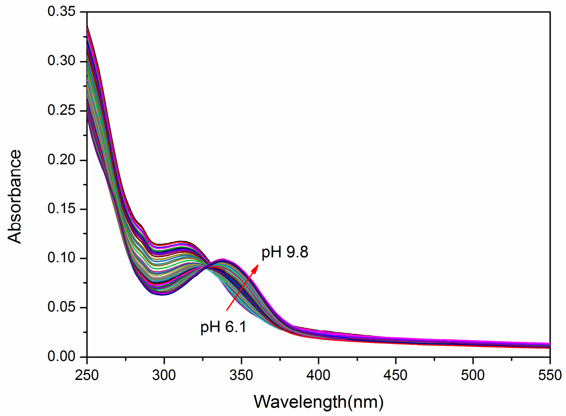

Fig. S15 Spectrophotometric titration curves for zinc(II) with L^1H_6, conditions: $[Zn^{2+}] = [L^1H_6] = 2 \times 10^{-5}$ M; $\mu = 0.10$ M KCl; $T = 298.2$ K; pH range = 6.1-9.8; 5.0 vol % methanol aqueous

solution.

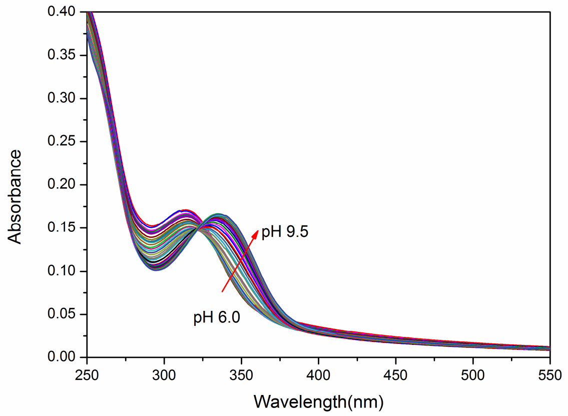

Fig. S16 Spectrophotometric titration curves for zinc(II) with L^2H_6, conditions: $[Zn^{2+}] = [L^2H_6] = 2 \times 10^{-5}$ M; $\mu = 0.10$ M KCl; $T = 298.2$ K; pH range = 6.0-9.5; 5.0 vol % methanol aqueous solution.

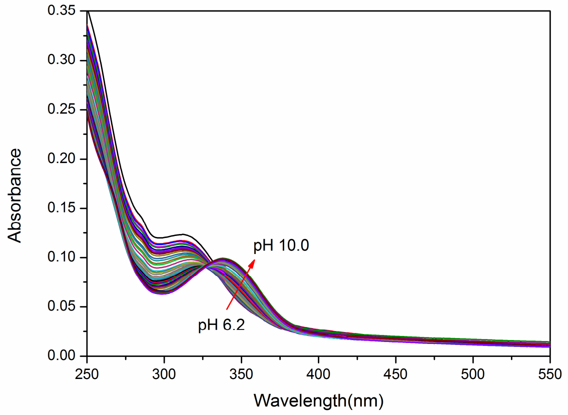

Fig. S17 Spectrophotometric titration curves for zinc(II) with L^3H_6, conditions: $[Zn^{2+}] = [L^3H_6] = 2 \times 10^{-5}$ M; $\mu = 0.10$ M KCl; $T = 298.2$ K; pH range = 6.2-10.0; 5.0 vol % methanol aqueous solution.

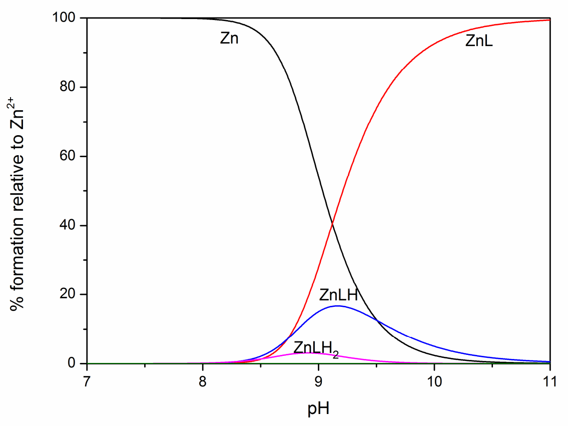

Fig. S18 Species distribution curves calculated for the zinc(II) complexes with ligand L^1H_6, the charge number are omitted for clarity, conditions: $[Zn^{2+}] = [L^1H_6] = 2 \times 10^{-5}$ M.

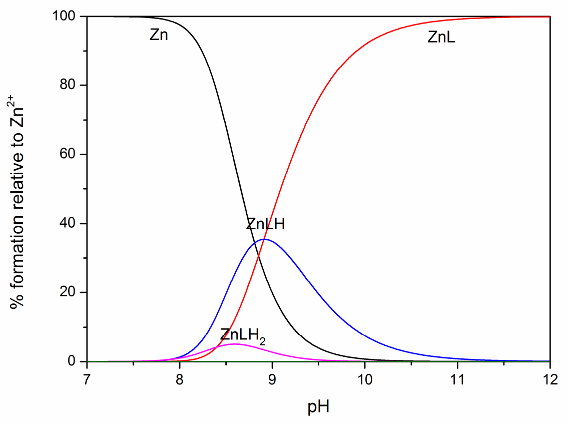

Fig. S19 Species distribution curves calculated for the zinc(II) complexes with ligand L^2H_6, the charge number are omitted for clarity, conditions: $[Zn^{2+}] = [L^2H_6] = 2 \times 10^{-5}$ M.

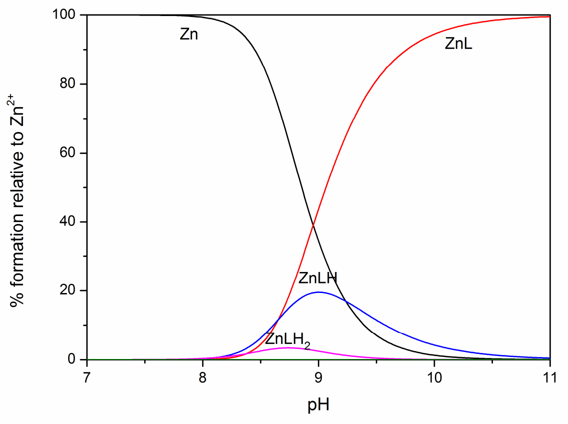

Fig. S20 Species distribution curves calculated for the zinc(II) complexes with ligand L^3H_6, the charge number are omitted for clarity, conditions: $[Zn^{2+}] = [L^3H_6] = 2 \times 10^{-5}$ M.

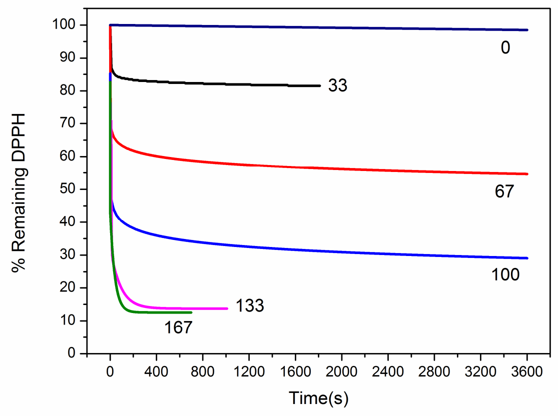

Fig. S21 The kinetic curves of antioxidant L^1H_6 with different concentrations mol AH/mol DPPH· ($\times 10^{-3}$).

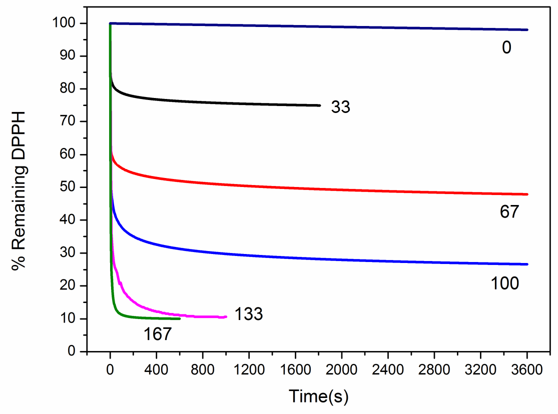

Fig. S22 The kinetic curves of antioxidant L^2H_6 with different concentrations mol AH/mol DPPH· ($\times 10^{-3}$).

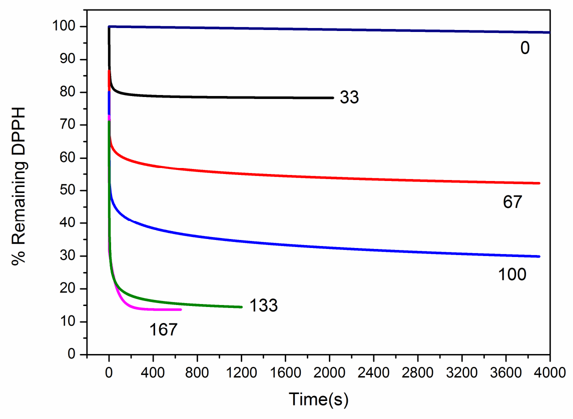

Fig. S23 The kinetic curves of antioxidant L^3H_6 with different concentrations mol AH/mol DPPH· ($\times 10^{-3}$).

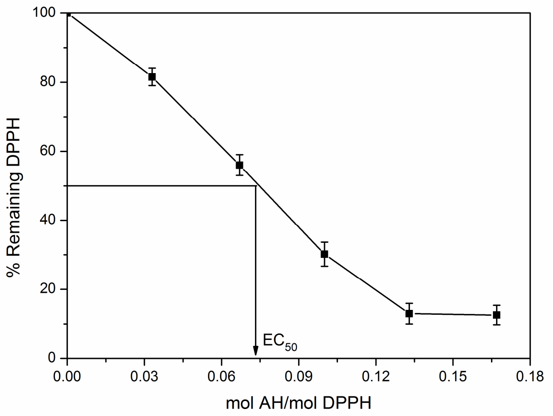

Fig. S24 The curve of remaining DPPH· against the molar antioxidant L^1H_6 to DPPH·.

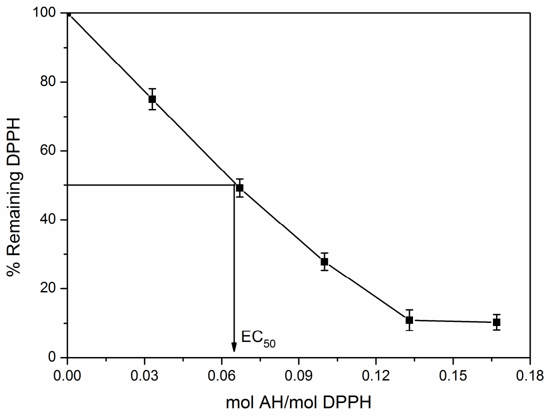

Fig. S25 The curve of remaining DPPH· against the molar antioxidant L^2H_6 to DPPH·.

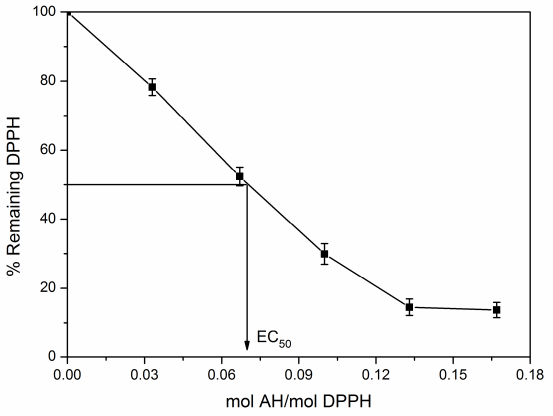

Fig. S26 The curve of remaining DPPH· against the molar antioxidant L^3H_6 to DPPH·.

References

[1] S. Salama, J. D. Stong, J. B. Neilands and T. G. Spiro, *Biochemistry*, 1978, **17**, 3781-3785.

[2] W. R. Harris, C. J. Carrano, S. R. Cooper, S. R. Sofen, A. E. Avdeef, J. V. McArdle and K. N. Raymond, *J. Am. Chem. Soc.*, 1979, **101**, 6097-6104.

[3] L. K. Charkoudian and K. J. Franz, *Inorg. Chem.*, 2006, **45**, 3657-3664.
